# Distinctive features and differential regulation of the *DRTS* genes of *Arabidopsis thaliana*

**DOI:** 10.1371/journal.pone.0179338

**Published:** 2017-06-08

**Authors:** Antonio Maniga, Stefania Ghisaura, Lara Perrotta, Maria Giovanna Marche, Rino Cella, Diego Albani

**Affiliations:** 1 Department of Agriculture, University of Sassari, Sassari, Italy; 2 Department of Science for Nature and Environmental Resources, University of Sassari, Sassari, Italy; 3 Department of Biology and Biotechnology, University of Pavia, Pavia, Italy; 4 Center of Excellence for Biotechnology Development and Biodiversity Research, University of Sassari, Sassari, Italy; McGill University, CANADA

## Abstract

In plants and protists, dihydrofolate reductase (DHFR) and thymidylate synthase (TS) are part of a bifunctional enzyme (DRTS) that allows efficient recycling of the dihydrofolate resulting from TS activity. *Arabidopsis thaliana* possesses three *DRTS* genes, called *AtDRTS1*, *AtDRTS2* and *AtDRTS3*, that are located downstream of three members of the sec14-like *SFH* gene family. In this study, a characterization of the *AtDRTS* genes identified alternatively spliced transcripts coding for AtDRTS isoforms which may account for monofunctional DHFR enzymes supporting pathways unrelated to DNA synthesis. Moreover, we discovered a complex differential regulation of the *AtDRTS* genes that confirms the expected involvement of the *AtDRTS* genes in cell proliferation and endoreduplication, but indicates also functions related to other cellular activities. *AtDRTS1* is widely expressed in both meristematic and differentiated tissues, whereas *AtDRTS2* expression is almost exclusively limited to the apical meristems and *AtDRTS3* is preferentially expressed in the shoot apex, in stipules and in root cap cells. The differential regulation of the *AtDRTS* genes is associated to distinctive promoter architectures and the expression of *AtDRTS1* in the apical meristems is strictly dependent on the presence of an intragenic region that includes the second intron of the gene. Upon activation of cell proliferation in germinating seeds, the activity of the *AtDRTS1* and *AtDRTS2* promoters in meristematic cells appears to be maximal at the G1/S phase of the cell cycle. In addition, the promoters of *AtDRTS2* and *AtDRTS3* are negatively regulated through E2F *cis*-acting elements and both genes, but not *AtDRTS1*, are downregulated in plants overexpressing the AtE2Fa factor. Our study provides new information concerning the function and the regulation of plant *DRTS* genes and opens the way to further investigations addressing the importance of folate synthesis with respect to specific cellular activities.

## Introduction

Cofactors derived by the tetrahydrofolate (THF), collectively named folates or vitamin B9, are essential for all organisms and are necessary for the addition or removal of one-carbon units in key reactions of various biochemical pathways (C1-metabolism). These reactions are crucial for the synthesis of a large number of compounds in many cellular processes, including amino acid and nucleic acid metabolisms. THF coenzymes are directly required for the synthesis of purines and thymidylate, for the interconversion of serine and glycine and for the synthesis of methionine (Met). Moreover, because Met is necessary for the formation of *S*-adenosyl-Met (Ado-Met), a major donor of methyl groups, the THF coenzymes in plants are indirectly involved in the synthesis of ethylene and polyamines, as well as in the production of a variety of molecules such as choline, chlorophyll and lignin, that require Ado-Met as a donor of methyl units [1,2]. In leaves of C3 plants, folates are known to support a huge metabolic flux concerning the photorespiratory process, where up to 30% of the folate pool participates in the THF-mediated conversion of glycine to serine in mitochondria [3]. Moreover, recent studies have also revealed important links between folates and the epigenetic control of gene expression, which is related to the Ado-Met involvement in DNA and histone methylation [4,5,6]. Additional roles of folates in iron—sulphur cluster metabolism have been described [7] and 5,10-methylene THF is known to be necessary for the synthesis of pantothenate (vitamin B5), which is essential for the the synthesis of CoA and ACP (acyl-carrier protein) involved in reactions of β-oxidation generating important signaling molecules such as indoleacetic, jasmonic and salicylic acids [8,9]. Because of the multiple and essential roles of THF cofactors, it is clear that the control of THF biosynthesis has important implications for plant growth and productivity, as confirmed by the embryo-lethal phenotype of biosynthetic mutants of *Arabidopsis thaliana* that are unable to produce functional folates [10,11].

Folates are tripartite molecules containing pterin, *p*-aminobenzoate (pABA) and glutamate moieties and are synthesized *de novo* by plants, fungi, most bacteria and protozoa. In plants, the biosynthesis of THF depends on the activity of enzymes that are localized in the cytosol, plastids and mitochondria [12]. Dihydropterin is synthesized from GTP in the cytosol, whereas p-ABA is synthesized in the plastids from chorismate. The final steps of THF synthesis occur in mitochondria in which dihydropterin and p-ABA are combined together with glutamate to produce the monoglutamate form of THF. In all organisms, folates occur mainly in the form of polyglutamylates derivatives that are obtained by the sequential addition of γ-linked glutamate residues to THF in a reaction catalysed by FPGS (folyl-polyglutamate synthetase). Once synthesized in the mitochondria, the monoglutamate form of THF is exported to the other cell compartments before the final glutamylation step. Glutamylation is essential to retain folates in a given compartment of the cell by increasing the anionic nature of folate coenzymes, thus impairing their diffusion through hydrophobic barriers [13].

Apart from its role as a carrier of one-carbon units, THF can also acts as a reducing agent. In particular, the synthesis of thymidylate, catalyzed by the enzyme thymidylate synthase (TS), requires N5,N10-methylene tetrahydrofolate to methylate and reduce deoxyuridine monophosphate (dUMP) to dTMP, yielding 7,8-dihydrofolate (DHF) as a secondary product. To enable efficient recycling of the resulting DHF, the activity of TS must be linked to the activity of dihydrofolate reductase (DHFR), the last enzyme of the biosynthetic pathway [1]. Although animals, fungi and bacteria possess monofunctional DHFR and TS enzymes, in plants and in protists DHFR and TS are part of a bifunctional enzyme (DRTS), a feature that favors substrate channeling and emphasizes the importance of a coordinated regulation of these activities. Thus, the DHFR domain of the bifunctional enzyme is involved in the reduction of DHF originating from either the *de novo* synthesis pathway (monoglutamate form) or the oxidation of THF by the TS activity (polyglutamate form) [1].

Plant *DRTS* genes have been described in Arabidopsis, carrot, soybean and maize [14–18], but additional *DRTS* sequences of other species are available through genomic and EST databases, including sequences from several primitive plants and algal species. All the DRTS proteins possess a conserved amino terminal DHFR region which is separated from the conserved carboxy terminal TS domain by a junctional region of variable sequence whose length has been shown to be critical for TS activity and domain-domain interaction of the bifunctional enzyme [19].

As the DHFR and TS activity are essential for the biosynthesis of nucleotides, analyses have focused on their importance in proliferating tissues or in tissues that are characterized by endoreduplication events. In situ hybridization analyses carried out in *Daucus carota* revealed that *DcDRTS* transcripts are particularly abundant in dividing cells of somatic embryos. In addition, northern blot hybridization experiments revealed a stronger accumulation of *DcDRTS* transcripts in proliferating suspension cells compared to cells in stationary phase or cells blocked with propyzamide [20]. In *Zea mays*, high expression of *ZmDRTS* was detected during early stages of kernel formation, exhibiting developmentally controlled endoreduplication, as well as in root tips, where cell division occurs, whereas low expression was found in the root elongation zone and leaves [17]. Also a recent investigation of the expression of the four *ZmDRTS* genes found in the maize genome revealed that all of them are maximally expressed at the beginning of kernel formation [18].

*Arabidopsis thaliana* possesses three *DRTS* genes, called *AtDRTS1*, *AtDRTS2* and *AtDRTS3*, that show a similar genomic organization and are located downstream of three members of the sec14-like *SFH* gene family, which suggests their origin from evolutionary genome duplications. The *AtDRTS1* and *AtDRTS2* genomic sequences have been published [14] and a gene model has been proposed for *AtDRTS3*, but information concerning the expression and the regulation of the Arabidopsis *DRTS* genes has not been reported so far. In this study, we describe a molecular characterization of the *AtDRTS* genes that reveals the existence of isoforms resulting from differential mRNA processing. Moreover, analyses of their expression and of their promoter activity reveal distinctive features of the three *AtDRTSs*, with different patterns of expression in both meristematic and differentiated cells. Although all three genes are variably expressed in the shoot apical meristems, only *AtDRTS1* and *AtDRTS2* are expressed in root apical meristems whereas *AtDRTS3* is characterized by a particularly strong expression in the root cap cells. The differential regulation of the *AtDRTS* genes is associated to distinctive promoter architectures and an intragenic region including the second intron of *AtDRTS1* is strictly required for its expression in root meristems. Finally, functional analyses of the E2F *cis* elements found in the promoter of *AtDRTS2* and *AtDRTS3* and evaluation of the accumulation of *AtDRTS* transcripts in transgenic plants overexpressing the AtE2Fa factor suggest that both *AtDRTS2* and *AtDRTS3*, but not *AtDRTS1*, are negatively regulated by E2F factors.

## Materials and methods

### Plant material and plant transformation

For germination and growth in aseptic conditions, wild type or transgenic *Arabidopsis thaliana* ecotype Columbia seeds were surface sterilized for 8/10 hours in 2% v/v PPM^®^ (Plant Preservative Mixture, Plant Cell Technology) supplemented with 50 mg/L magnesium salts (MgSO_4_). Seeds were imbibed for 2 days in 0,1% agarose at 4°C in the dark and then germinated on petri plates containing MS salts (Duchefa Biochemie), supplemented with Sucrose (10g/l) and Phyto agar (8g/l) (Duchefa Biochemie) and incubated in a growth cabinet at 22°C under long day conditions of 16 h of light and 8 h of dark.

The transgenic *Arabidopsis* lines used in this study were generated by the floral dip method [21] using *Agrobacterium tumefaciens* GV3101/pMP90 and EHA105 strains. Transformed T1 and progeny plants were selected on MS plates containing the resistance antibiotic (Hygromycin, 10mg/l). At two weeks of age, the resistant plants were transferred to recovery plates and grown for one more week in aseptic conditions without the selection agent before transferring them to soil. Plants were grown to maturity in growth cabinets set at long day conditions of 16 h of light (22±3°C) and 8 h of dark (22±3°C), with 70% relative humidity. Single insertion lines were identified by quantitative PCR reactions with leaf DNA and confirmed by segregation of hygromycin resistance in the T2 progeny.

### Generation of promoter constructs

For the production of the SFH7/DRTS1, SFH1/DRTS2 and SFH3/DRTS3 dual reporter constructs, the promoter regions of *AtDRTS1* (reported in BAC clone F16F14), *AtDRTS2* (described in BAC clone T4L20) and *AtDRTS3* (found in BAC clone F2G1) were amplified from *Arabidopsis* genomic DNA using primers designed to amplify the entire intergenic region comprised between the ATG codons of the *AtDRTS* and *AtSFH* genes. For the SFH1/DRTS2 construct the fragment was extended up to the second in frame ATG codon of *AtDRTS2*, which is located in the fourth exon of the gene. The *AtDRTS1/AtSFH7* intergenic region was amplified using the primers F16F4 (which creates a terminal *Xba*I site over the start codon of *AtSFH7*) and F16F2 (which creates a terminal *Bam*HI site downstream of the ATG of *DRTS1*). To isolate the *AtDRTS2*/*AtSFH1* intergenic region, the primers used were T4L1 (which introduces overlapping *Xba*I and *Bgl*II sites over the ATG of *AtSFH1*) and T4L2 (which contains the original *Nco*I site found over the ATG codon of *AtDRTS2*). Finally, the *AtDRTS3*/*AtSFH3* intergenic region was amplified using the primers F2G1 (which creates a *Pst*I site over the start codon of *AtSFH3*) and F2G2 (which introduces a *Nco*I site downstream of the ATG of *AtDRTS3*). The PCR amplifications were carried out using high fidelity Pfx Taq polymerase (Invitrogen) and the resulting DNA fragments were cloned into pBS-KS or pGEM-T Easy plasmids and sequenced to verify the fidelity. The promoter regions were then inserted into the pCambia 1301 binary vector, cloning the *AtDRTS* promoters upstream to the *GUS* reporter gene, giving rise to the pCAMBIA-F16F14, pCAMBIA-T4L20 and pCAMBIA-F2G1 plasmids. To generate the SFH/DRTS dual reporter constructs, in which the *AtSFH* promoters direct the expression of the *eqFP*611 reporter gene, the *eqFP611* cDNA was amplified from the pQ32 vector [22] and inserted upstream to the 35S terminator sequence into the pFF19 vector [23]. The DNA fragment containing the *eqFP*611 and 35S-ter sequences was then excised and inserted into the pCAMBIA-F16F14, pCAMBIA-T4L20 and pCAMBIA-F2G1 plasmids to give rise to the SFH7/DRTS1, SFH1/DRTS2 and SFH3/DRTS3 dual reporter constructs. For the SFH7/DRTS1i2 construct, which includes the second intron of *AtDRTS1*, the PCR was performed using the primers F16F4 (which anneals next to the *AtSFH7* start codon and creates a terminal *Xba*I site) and F16F5 (which anneals at the beginning of the *AtDRTS1* third exon and creates a *Bam*HI site with a correct reading frame allowing translational fusion with the *GUS* coding region).

Mutations of the E2F binding sites in the cloned *AtDRTS2* and *AtDRTS3* promoter fragments were created by PCR. The DNA portions flanking both sides of the E2F site were amplified from the cloned genomic fragments using universal primers (M13-FW or M13-RV), annealing next to the polylinker of the plasmids, in combination with specific primers that anneal over the internal E2F site and create an *Eco*RI restriction site in place of the SSCGSS sequence (T4L6 and T4L7 for AtDRTS2-ΔE2F promoter, F2G3 and F2G4 for AtDRTS3-ΔE2F promoter). After joining the two resulting PCR fragments at their *Eco*RI ends, the mutated promoters were introduced into the SFH1/DRTS2 and SFH3/DRTS3 constructs, replacing the wild type *AtDRTS2* and *AtDRTS3* promoter sequences, to give rise to the SFH1/DRTS2ΔE2F and SFH3/DRTS3ΔE2F constructs. All the primer sequences are detailed in S1 Table.

### Generation of *AtE2Fa* overexpressing lines

To generate a vector for the overexpression of *AtE2Fa*, an expression cassette was obtained inserting the *AtE2Fa* cDNA fragment in the polylinker of the pFF19 vector [23], downstream of the double CaMV 35S promoter and upstream of the 35S terminator sequence. The entire cassette was then excised as a *Hin*dIII-*Eco*RI fragment and cloned into the pCambia 1304 binary vector which contains the GUS reporter gene under the control of the CaMV 35S promoter. Following *Agrobacterium*-mediated transformation by the floral dip method and selection of the transformants, DNA from the transformed plants was analysed by qPCR to identify single insert lines and their progeny was selected on hygromycin in order to identify the homozygous AtE2Fa^OE^ lines.

### Nucleic acids extraction and qRT-PCR analyses

Genomic DNA from transformed T1 plants was isolated using a modified CTAB protocol [24]. About 100 mg of leaf tissue were homogenated in 500 μl of CTAB Extraction Buffer (2% cetyl trimethylammonium bromide, 1% polyvinyl pyrrolidone, 100 mM Tris-HCl pH 7.5, 1.4 M NaCl, 20 mM EDTA). After incubation for 30 min at 65°C, the gDNA was purified using chloroform/isoamyl alcohol (24:1) and precipitated in isopropanol. The DNA pellets were washed in 70% ethanol and resuspended in 50 μl of TE buffer.

Total RNA extractions were performed using the Qiagen RNeasy mini-kit. The RNA samples were digested with Dnase I during the extraction using the Qiagen RNase-free DNase set. RNA concentration and quality have been evaluated by spectrophotometry using A260/A280 ratio and by electrophoresis on denaturing formaldehyde gel. For qRT-PCR analyses, 1μg of RNA has been reverse transcribed using the Invitrogen SuperScript^®^ III Reverse Transcriptase with a combination of examers and oligo dT primers. The qRT-PCR analyses were repeated three times using independent biological replicates. Quantitative PCR was performed on the BioRad iCycler iQ ^™^, using the Qiagen QuantiTect SYBR^®^ Green PCR Kit. For each sample triplicate PCR reactions have been performed following the manufacturer's recommended amplification conditions. For all the analyses the amplification 18S RNA has been used as a reference for normalization. Quantification was calculated following the ΔΔ^Ct^ method [25]. The PCR primers were designed using the Primer3 online software (http://primer3.ut.ee) and all their sequences are detailed in S2 Table.

### GUS assays

Histochemical detection of GUS activity was performed on transgenic plants using 5-bromo-4-chloro-3-indolyl-β-D-glucuronide (X-Gluc) [26]. Plants at different developmental stages were incubated overnight at 37°C in the GUS solution (50 mM pH 7 phosphate buffer, 1 mg/mL X-Gluc, 1 mM potassium ferricyanide). After staining, to avoid interference, chlorophyll was removed treating the samples in 70% ethanol.

For quantitative analyses, the level of GUS activity was detected fluorimetrically using the fluorogenic substrate MUG (4-methyl-umbelliferyl—glucuronide). Seedlings of the same developmental stage were ground in GUS extraction buffer (50 mM NaPO_4_ pH 7, 10 mM EDTA, 0.1% Triton, 0.1% Sodium Lauryl Sarcosine, 10 mM β-Mercaptoethanol). An aliquot of the extracts was added to 300 μl of assay buffer (50 mM NaPO_4_ pH 7, 10 mM EDTA, 0.1% Triton, 0.1% Sodium Lauryl Sarcosine, 10 mM β-Mercaptoethanol, 1mM MUG) and the reactions were incubated at 37°C. At different time points, 100 μl of the reaction mix were added to 900 μl of stop buffer (0.2 M Na_2_CO_3_) and the amount of 4MU produced was measured using a fluorimeter (BioRad). The protein concentration of each extract was assayed using the Bradford method [27] to allow calculation of the specific GUS activities.

### Treatments with cell cycle inhibitors

To perform the treatments with cell cycle inhibitors, 30 seeds of selected homozygous transgenic lines harbouring the SFH7/DRTS1i2, SFH1/DRTS2 or SFH1/DRTS2ΔE2F constructs were imbibed in sterile water alone (as control) or in water containing 5 μg/ml aphidicolin (Fisher Scientific) or 5 mg/ml colchicine (Apollo Scientific). After 72 h of imbibition in growth chamber at 22°C under a regimen of 16 h of light at and 8 h of dark, proteins were extracted and fluorimetric assays of GUS activity were performed.

## Results

### Molecular characterization of the *AtDRTS* genes

The genome of *Arabidopsis thaliana* contains three *DRTS* genes called *AtDRTS1*, *AtDRTS2* and *ATDRTS3* (also named *THY1*, *THY2* and *THY3)*. The *AtDRTS1* gene, annotated as At2g16370 in the TAIR database, is located on the minus strand of chromosome 2. According to the proposed gene model, the gene extends 2774 bp, from position 7088865 to 7091639, and is divided into 10 exons that give rise to a transcript of 1924 bp. The predicted ATG start codon is located in the second exon and the resulting coding region translates into a protein of 519 aa with a MW of 58.1 KDa. The *AtDRTS2* gene, annotated as At4g34570, based on the gene model spans 3310 bp on the minus strand of chromosome 4, from position 16511006 to 16514316 and contains 12 exons resulting in a 1926 bp transcript. The ATG start codon is found at the end of the second exon and the predicted coding region translates into a protein of 565 aa with a MW of 63.2 KDa. Finally, the *AtDRTS3* gene, with annotation At2g21550, is located on the plus strand of chromosome 2 and the proposed gene model is divided into 10 exons and extends 2980 bp, from the ATG triplet at position 9234289 to the TAA stop codon at 9237269. The predicted transcript includes an open reading frame of 1476 bp that is expected to code for a protein of 492 aa with a MW of 55,3 KDa.

**Fig 1:**
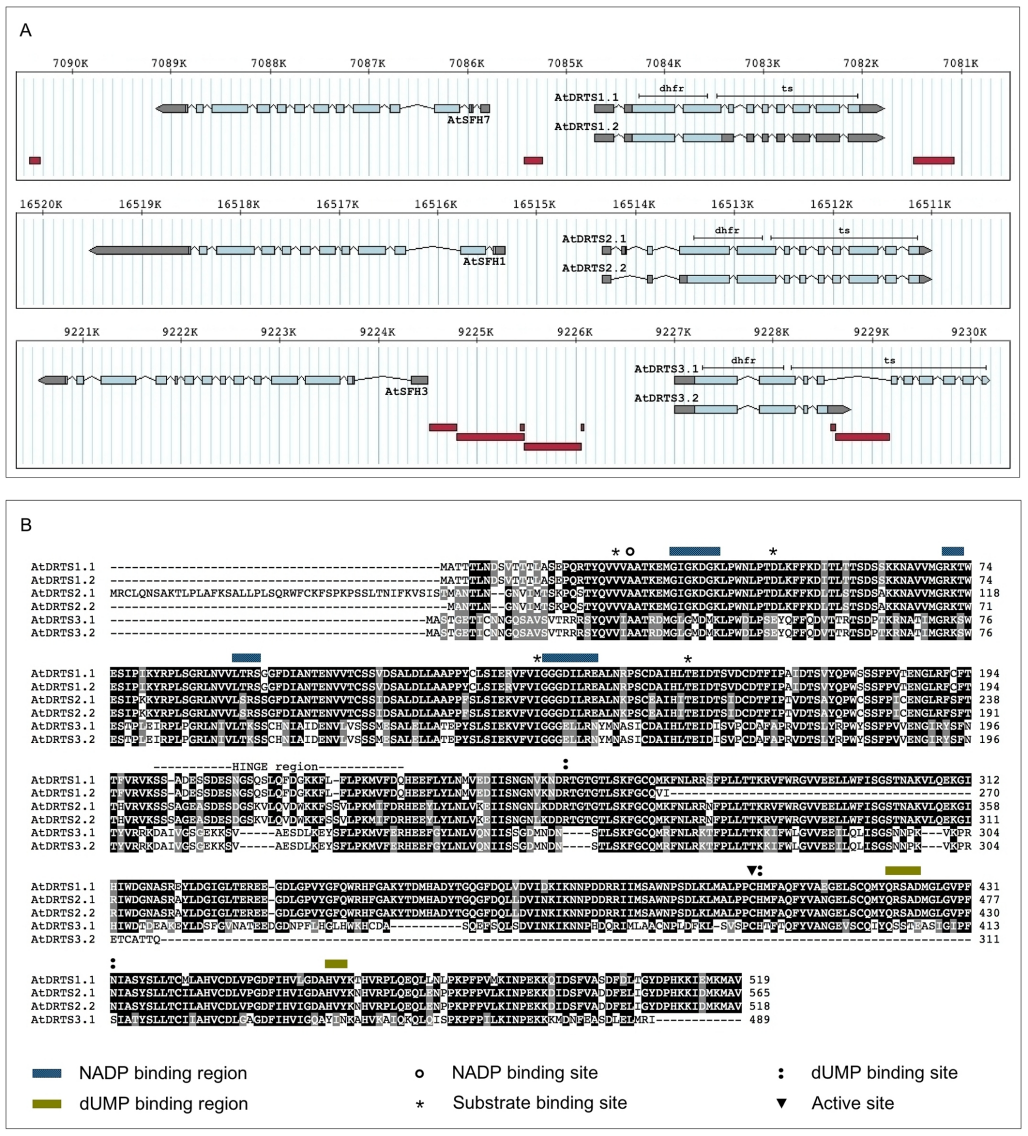
*AtDRTSs* gene structure and protein isoforms. (A) Genomic organization of the *AtDRTS* gene paralogs and of the upstream *AtSFH* members. The exons are indicated as boxes with the UTR regions shown in gray and the coding portions in light blue. The portions corresponding to the DHFR and TS domains are indicated above the structure of the longest isoforms. The position of transposable elements is shown as dark red boxes below the gene structures. (B) Amino acid sequence comparison of the AtDRTS isoforms. The functional sites are indicated as described in the legend.

As shown in Fig 1A, for all three *AtDRTS* genes the region encoding the DHFR domain is located in the two largest exons, whereas the TS domain is spanning over as many as 8 (*AtDRTS1* and *AtDRTS2*) or 9 exons (*AtDRTS3*). Moreover, the conserved genomic organization of the *AtDRTS* loci reveals that all three genes are downstream to members of the sec14p-like *SFH* (Sec Fourteen Homologues) gene family, which are oriented in opposite direction with respect to the *AtDRTS* genes. Thus, the intergenic region separating the *AtDRTS* and the *AtSFH* genes contains the promoters of both genes. The *AtSFH* gene family is composed of 14 members that code for a group of phosphatidylinositol transfer proteins (PITP)s with physiological functions related to lipid metabolism, phosphoinositide mediated signalling and membrane trafficking [28]. In chromosome 2, *AtSFH7* (At2g16380) is upstream to *AtDRTS1* and *AtSFH3* (At2g21540) is upstream to *AtDRTS3*, whereas in chromosome 4 *AtSFH1* (At4g34580), also known as *COW1*, is the *SFH* gene located upstream to *AtDRTS2*. As also shown in Fig 1A, transposon-like elements are inserted in the *AtSFH7*/*AtDRTS1* and *AtSFH3*/*AtDRTS3* intergenic regions as well as in the fourth intron of the *AtDRTS3* gene.

Remarkably, database sequences and experimental analyses of the transcripts reveal the existence of at least two isoforms of each *AtDRTS* gene, some of which are potentially coding for truncated proteins lacking most of the TS domain (Fig 1A and 1B). In this respect, although the genomic structure of *AtDRTS1* and *AtDRTS2* was supported by cDNA sequences, cDNA clones confirming entirely the predicted gene model of *AtDRTS3* have not been reported. The only *AtDRTS3* cDNA sequence available in databases (accession number BX820604) confirms the predicted position of the first three exons but extends the fourth exon into part of the following intron, that contains a transposon-like element in which an in frame stop codon interrupts the coding sequence (Fig 1A). Thus, it appears that the presence of the transposon-like element in the fourth intron of *AtDRTS3* can cause premature termination of the primary transcripts and yields a mRNA retaining part of the fourth intron and coding for a protein of 311 aa, with predicted mass of 35 kDa, that is expected to possess only DHFR activity. However, although *AtDRTS3* cDNA sequences including all the TS coding region have not been reported, microarray analyses (ATH1 Probe Set 263546_at) suggested the expression of transcripts spanning over the 3' end of the putative *AtDRTS3* gene model. To verify whether full length *AtDRTS3* transcripts corresponding to the proposed gene model can be actually produced, RT-PCR reactions were performed using a forward primer that overlaps the ATG start codon in the first exon and a reverse primer that overlaps the predicted TAA terminating triplet, which is located in the tenth exon of the gene. These RT-PCR reactions were performed with high fidelity Taq polymerase, using retrotranscribed RNA isolated from Arabidopsis seedlings, and allowed the amplification of a cDNA containing the entire predicted coding region of the *AtDRTS3* gene model. Although the resulting sequence did not show any nucleotide change compared to the exonic sequences reported in the TAIR database, the 5' splicing site of the sixth intron occurs 9 bp upstream of the predicted one and yields a mRNA that is coding for an AtDRTS3 protein of 489 aa, with a predicted MW of 54.9 kDa, that is slightly smaller than the protein proposed by the gene model. Thus, in spite of the transposon element in the fourth intron, it appears that the *AtDRTS3* gene can give rise to a full length transcript encoding a bifunctional DHFR/TS protein (Fig 1B). Based on these results, we called *AtDRTS3*.*1* the large isoform, corresponding nearly exactly to the gene model, and *AtDRTS3*.*2* the smaller one, terminating at the fourth intron and coding for a protein that lacks most of the C-terminal TS domain. Interestingly, a similarly truncated protein appears to be encoded also by an alternatively spliced transcript of *AtDRTS1* corresponding to the cDNA clone reported in databases with the accession number BX820156. This isoform, which we named *AtDRTS1*.*2*, retains the third intron, containing an in frame stop codon, and the interrupted open reading frame is predicted to code for a protein of 270 aa, with a predicted MW of 30 kDa. Compared to the 519 aa long AtDRTS1.1 protein of 58.1 kDa, the AtDRTS1.2 isoform lacks most of the TS domain and, similarly to AtDRTS3.2, is expected to display only DHFR activity (Fig 1B). Also concerning the *AtDRTS2* gene two isoforms have been detected, but both are coding for bifunctional DHFR/TS proteins (Fig 1A). In this respect, 5’RACE analyses performed in our laboratory revealed the existence of alternatively spliced *AtDRTS2* transcripts lacking the second exon that contains the proposed ATG start codon of the gene. Its absence in the alternative transcripts results in the translation of a 518 aa long isoform, which we named AtDRTS2.2, that begins from the in-frame ATG codon located in the fourth exon that was originally proposed as a start codon by Lazar et al. [14]. The predicted mass of AtDRTS2.2 is 57.9 kDa whereas AtDRTS2.1 possesses a N-terminal extension of 47 aa that increases the MW to 63.2 kDa.

As described in Table 1, a comparison of the larger isoforms of the AtDRTS proteins revealed a close homology between AtDRTS1 and AtDRTS2, showing over 86% amino acid identity, whereas AtDRTS3 appears to have partially diverged, with 56,8 and 57,4% identity to AtDRTS1 and AtDRTS2 respectively. This divergence is further highlighted by comparison of the highly variable hinge region separating the two functional domains, that shows as much as 64,7% identity between AtDRTS1 and AtDRTS2 whereas for AtDRTS3 shows only 33,3 and 38,7% identity compared to AtDRTS1 and AtDRTS2. Remarkably, as shown in S1 Fig, compared with the DRTSs of other angiosperms described in literature and databases, AtDRTS3 groups together with a subset of the plant DRTS sequences. Moreover, although the cysteine corresponding to the active site in the TS domain is conserved in all three AtDRTS large isoforms (Fig 1B), nearly all the substrate binding sites are perfectly conserved between AtDRTS1 and AtDRTS2 but several amino acid substitutions characterize most of the substrate binding sites of AtDRTS3 and could reflect functional peculiarities of this protein.

**Table 1:** Percent identity matrix of the AtDRTS large isoforms and of their hinge region (H).

	**AtDRTS1**	**AtDRTS2**	**AtDRTS3**	**AtDRTS1/H**	**AtDRTS2/H**	**AtDRTS3/H**
AtDRTS1	100.00					
AtDRTS2	86.85	100.00				
AtDRTS3	56.82	57.41	100.00			
AtDRTS1/H	-	-	-	100.00		
AtDRTS2/H	-	-	-	64.71	100.00	
AtDRTS3/H	-	-	-	33.33	38.71	100.00

The Matrix was created by Clustal2.1 on sequences aligned using the T-Coffee program (http://www.ebi.ac.uk/Tools/msa/tcoffee/).

Compared to AtDRTS2.1, the AtDRTS2.2 isoform lacks the first 47 aa and could potentially lack a signal peptide or could possess an amino-terminal region allowing a different organellar targeting of the enzyme. To investigate this possibility, predictions of the subcellular localization of the AtDRTS isoforms were performed with 10 different platforms available online. As shown in Table 2, AtDRTS1.1 was predicted to be cytosolic by 8 of the softwares while the truncated AtDRTS1.2 isoform was predicted to be cytosolic by only 5 of the platforms and additional predictions, including cell membrane, chloroplast, and extracellular locations, were proposed by some of the programs. Interestingly, the localization of AtDRTS2.1, which possesses a N-terminal extension that is absent in the other AtDRTSs, was predicted mostly as plastidial and/or mitochondrial whereas the smaller AtDRTS2,2 isoform was largely predicted as cytosolic. For AtDRTS3.1 a prevalence of cytosolic over plastidial localization was reported while the truncated AtDRTS3.2 isoform was predicted more as plastidial or membrane bound rather than cytosolic. Thus, different subcellular localizations of the AtDRTS proteins and of some of their isoforms are likely to occur.

**Table 2:** Predicted subcellular localization of the AtDRTS isoforms.

	AtDRTS1.1	AtDRTS1.2	AtDRTS2.1	AtDRTS2.2	AtDRTS3.1	AtDRTS3.2
iPSORT	PL	PL	M	PL	PL	PL
SubLoc	CY	EX	CY	CY	CY	NU
WoLFPSORT	CY	NU/CY	M/PL	M/PL	PL	PL
CELLO	CY	PM	M/PL	CY	PM	PM
EuLoc	CY	PM	CY	CY	CY	NU/PM
iLoc-Plant	NU	CY	NU	NU	CY	CY
PSI predictor	CY	CY/PL	M/PL	CY	CY/PL	PM/PL
PProwler	CY	CY	M	CY	CY	CY
YLoc	CY	EX	PL	CY	NU	CY
Plant-mPLoc	CY	PM/PL/CY/M/PX	CY/PL	CY	CY	PM/PL
**CONSENSUS**	**CY**	**CY**	**PL/M**	**CY**	**CY**	**PL/PM**

The AtDRTS sequences were analysed online with the following platforms: iPSORT (http://ipsort.hgc.jp), SubLoc (www.bioinfo.tsinghua.edu.cn/SubLoc/eu_predict.htm), WoLFPSORT (https://wolfpsort.hgc.jp), CELLO (http://cello.life.nctu.edu.tw), EuLoc (http://euloc.mbc.nctu.edu.tw), iLoc-Plant (http://www.jci-bioinfo.cn/iLoc-Plant), PSI predictor (http://bis.zju.edu.cn/psi), Pprowler(http://bioinf.scmb.uq.edu.au:8080/pprowler_webapp_1-2), Yloc (http://abi.inf.uni-tuebingen.de/Services/YLoc/webloc.cgi), Plant-mPLoc (http://www.csbio.sjtu.edu.cn/bioinf/plant-multi). The consensus indicates the most common predicted localization(s). CY, cytosol; PL, plastid; M, mitochondrion; NU, nucleus; PX, peroxisome; PM, plasma membrane; EX, extracellular.

### The *AtDRTS* genes are differentially expressed in both meristematic and differentiated tissues

**Fig 2:**
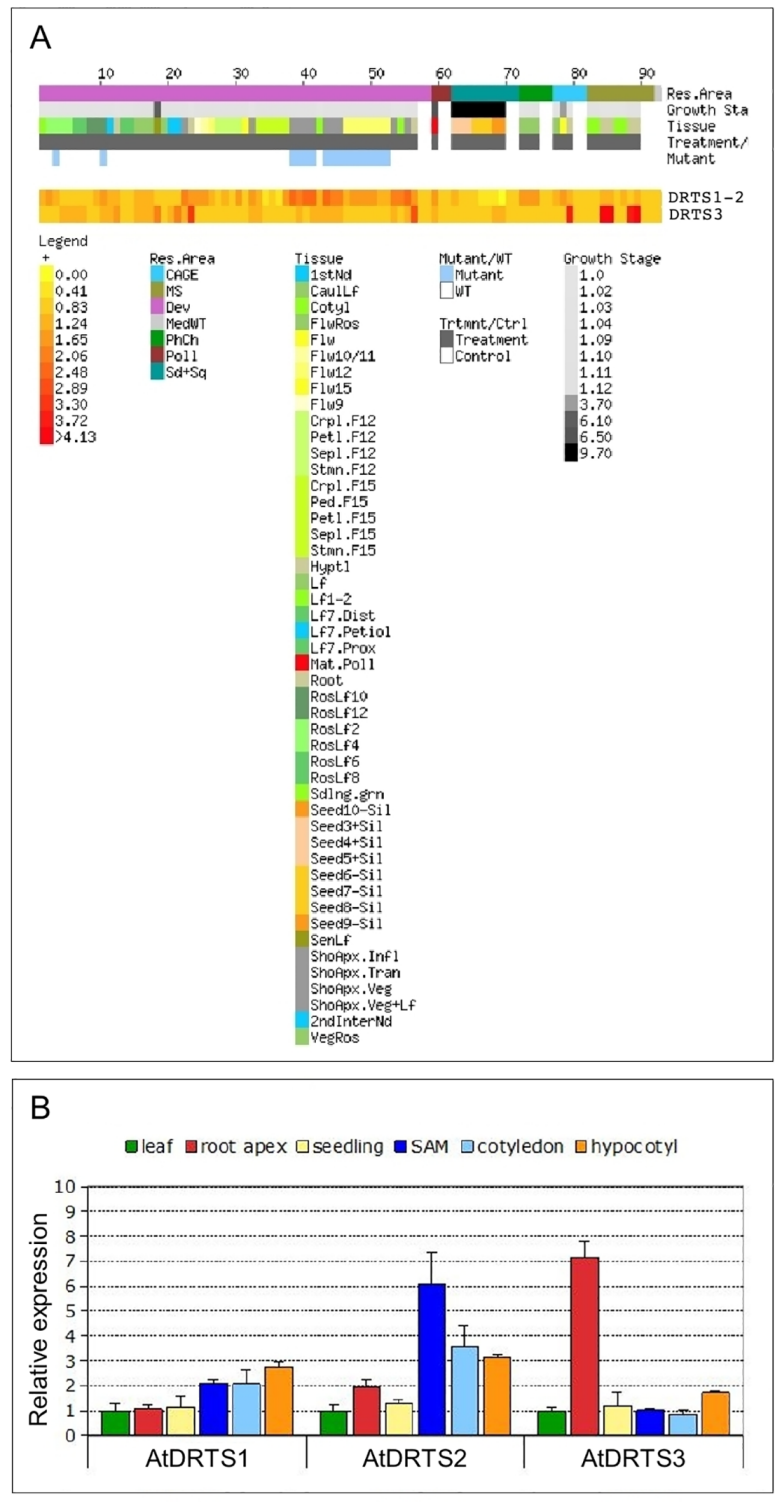
Analysis of the expression of the *AtDRTS* genes. (A) E-Northern analysis of the expression of the *AtDRTS* transcripts revealed by microarray data. Heat maps showing the expression levels of the *AtDRTS1/AtDRTS2* common gene set and of *AtDRTS3* across different samples were generated using the Expression Browser tool of the Botany Array Resource (BAR) (http://bar.utoronto.ca/). (B) qRT-PCR analysis of the relative expression levels of the *AtDRTSs* in representative organs compared to leaves. The qRT-PCR analyses were repeated three times using independent biological replicates and quantification was normalized to 18S RNA levels. The bars show standard errors.

Partial expression analyses of maize, carrot and soybean *DRTS* genes revealed a predominant expression of these genes in proliferating or endoreduplicating cells, in line with the crucial role played by thymidylate synthase in DNA synthesis. However, the THF produced by DHFR activity is required for several important cellular pathways and substantial expression of the *DRTS* genes is expected to occur in other cellular contexts as well. In this respect, the results of microarray analyses which are reported at the *Arabidopsis* eFP browser of the Bio-Array Resource (BAR) website (http://www.bar.utoronto.ca/efp/cgi-bin/efpWeb.cgi; [29]) and at the Genevestigator V3 web tool (https://www.genevestigator.ethz.ch/gv/index.jsp; [30]) suggest an expression of the *AtDRTS* genes in different plant organs, including also tissues in which cell proliferation or endoreduplication are not likely to occur (S2 Fig). These expression data are related to experiments performed using the Affimetrix ATH1 array in which *AtDRTS3* is represented by a specific probe set (263546_at) whereas *AtDRTS1* and *AtDRTS2* transcripts are hybridizing to a single probe set (263601_s_at) and their individual patterns of expression is not distinguishable. Nevertheless, as described in Fig 2A, the expression of the *AtDRTS1*/*AtDRTS2* couple and of *AtDRTS3* appear to be very distinctive. More specifically, the strongest signal for the *AtDRTS1*/*AtDRTS2* probe set was detected in the shoot apex and in seeds 24 hours after imbibition, whereas the *AtDRTS3* expression level is reported to be particularly strong in the root cap (S2 Fig).

To further investigate the expression of the AtDRTS genes and to verify whether, in addition to *AtDRTS3*, also *AtDRTS1* and *AtDRTS2* can show distinctive patterns of expression, qRT-PCR analyses were performed on Arabidopsis seedlings and organs. To discern the expression of the *AtDRTSs* in meristematic versus differentiated cells, the analyses were conducted with RNA isolated from root and shoot apices, as well as leaves, hypocotyls and cotyledons. The relative level of expression of the *AtDRTSs* in the various organs compared to the leaves was calculated by the ΔΔ^Ct^ method [25] and is reported in Fig 2B. These results reveal a remarkably higher expression of *AtDRTS3* in the root apex compared to the other organs, which agrees with the high level of expression detected in root caps by microarray analyses. A slight upregulation of *AtDRTS3* occurs also in hypocotyls, whereas similar levels of expression compared to the leaves are detected in seedlings, cotyledons and shoot apices. Concerning the expression of *AtDRTS1* and *AtDRTS2*, distinctive patterns were detected which, in agreement with the microarray analyses, reveal an upregulation of both genes in shoot apices compared to the leaves. In particular, the expression of *AtDRTS2* appears to be maximal in shoot apices and is clearly upregulated also in cotyledons and hypocotyls, as well as in the root apices to a lower extent. Conversely, *AtDRTS1* shows the strongest expression in hypocotyls and is clearly upregulated also in shoot apices and cotyledons, but shows similar levels of expression in the root apices and in leaves. The expression of all the *AtDRTS* genes in the shoot apex is likely correlated to different extents with cell proliferation and, at least for *AtDRTS1* and *AtDRTS2*, this correlation probably occurs in the root apex as well. The strong and variable expression of all three *AtDRTS* genes observed in differentiated tissues could be linked in part to cellular endoreduplication, but is likely to reflect also the involvement of the AtDRTS proteins in other cellular processes.

### The *AtDRTS* promoters are differentially active and *AtDRTS1* is controlled also by intragenic regions

**Fig 3:**
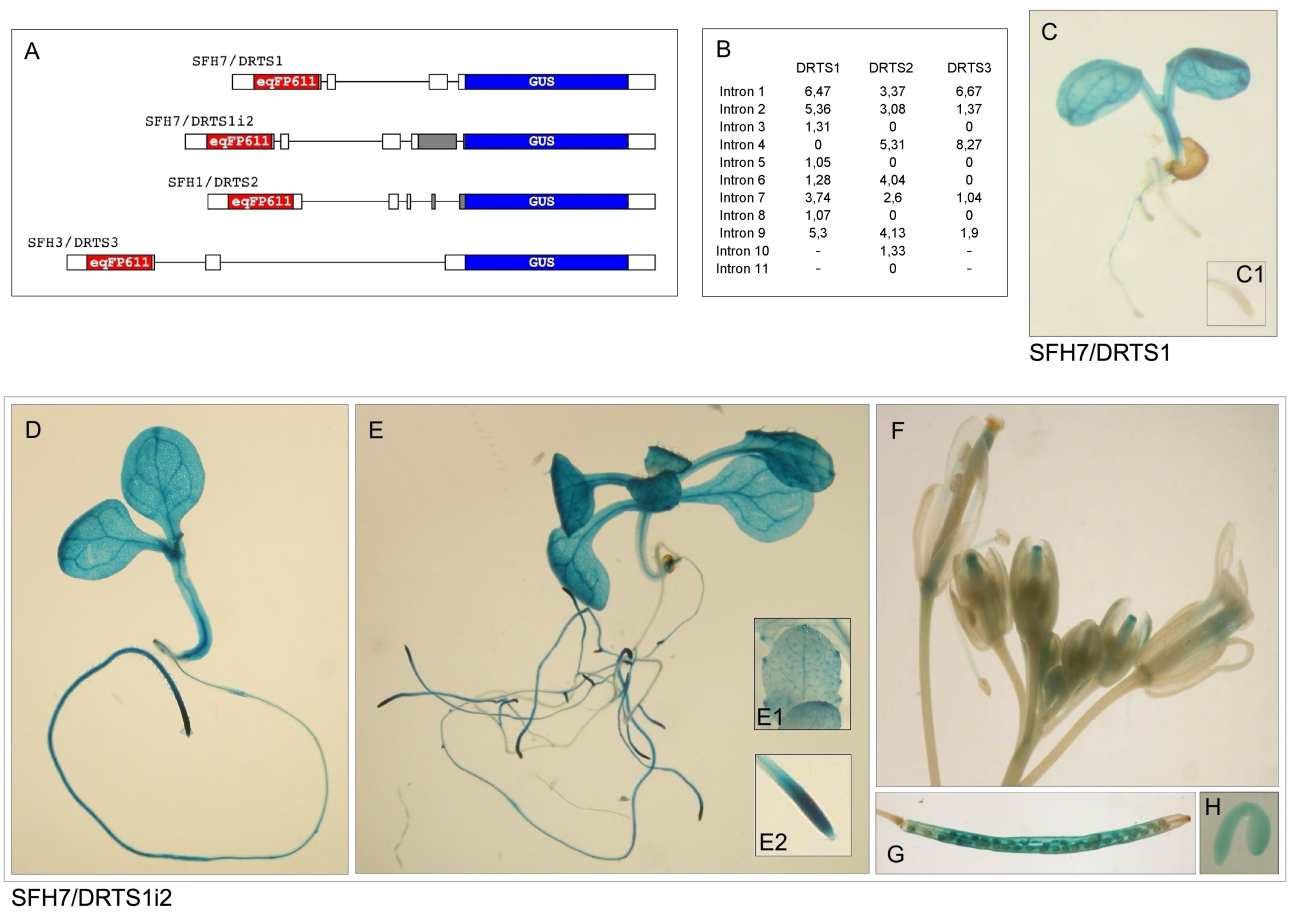
The activity of the *AtDRTS1* promoter in apical meristems is controlled by an intragenic region that includes the second intron of the gene. (A) Schematic representation of the dual reporter constructs used to test the activity of the divergent *AtDRTS* and *AtSFH* promoters. The *uidA* gene coding for the GUS protein is under the control of the *AtDRTS* promoters whereas the gene encoding the red fluorescent protein eqFP611 is controlled by the *AtSFH* promoters. (B) IMeter analysis of the introns of the *AtDRTS* genes revealing high scores of both the first and second intron of *AtDRTS1*. (C to H) Histochemical localization of GUS activity in transgenic Arabidopsis plants carrying different AtDRTS1 promoter constructs. (C) Localization of GUS accumulation in a one-week-old seedling of the SFH7/DRTS1 lines that reveal the inability of the *AtDRTS1* 5’ flanking region to drive expression in the RAM, magnified in the inset (C1). (D to H) Localization of GUS activity in lines carrying the SFH7/DRTS1i2 construct that includes the second intron of *AtDRTS1*. One-week-old (D) and two-week-old (E) seedlings showing strong activity of the *AtDRTS1* regulatory region in hydathodes and RAM, as highlighted in the insets (E1) and (E2). The activity of the DRTS1i2 promoter construct is clearly detected also in developing stigmas (F), siliques (G) and mature embryos (H).

To define more precisely the patterns of expression of the *AtDRTS* genes, the activity of their promoters was investigated in transgenic Arabidopsis plants. Considering that the intergenic region upstream to the *AtDRTSs* contains also the promoter of the divergent *AtSFH* genes, dual reporter constructs were assembled in which, as shown in Fig 3A, the DRTS promoter is controlling the expression of the *GUS* reporter gene while a gene coding for the red fluorescent protein eqFP611 [22] is placed under the control of the SFH promoter. Moreover, the *AtSFH1* and *AtSFH3* promoters have been already shown to be strongly active in roots and pollen respectively [31,32] and their presence can allow a quality control of the corresponding constructs. The genomic fragments of each intergenic region, spanning from the start codon of the *AtSFH* gene to the ATG codon of each *AtDRTS* gene, were amplified by PCR using high fidelity Taq polymerase and used for the production of the dual reporter constructs. More precisely, because our analysis of the *AtDRTS* isoforms revealed evidence of an alternative splicing of *AtDRTS2* that removes the second exon containing the first ATG of the gene, the genomic fragment amplified for the *AtDRTS2* promoter construct was extended up to the second in frame ATG, which is located in the fourth exon of the gene model. All these dual promoter constructs, called SFH7/DRTS1, SFH1/DRTS2 and SFH3/DRTS3, contain the 5’ untranslated region of the genes, which in several cases has been shown to be important for the correct activity of the promoters. Performing Agrobacterium-mediated transformation, 30 to 36 T1 transformants were obtained with each of the construct. The primary transformants were grown to maturity to allow seeds setting and quantitative PCR reactions with leaf DNA allowed the identification of single insertion lines. Six to eight single insertion lines, confirmed also by segregation of hygromycin resistance in the T2 progeny, were obtained from each transformation. Histochemical GUS assays, as well as fluorescence analyses for the detection of the eqFP611 protein, were performed on the T2 progeny of every line. Consistent results were obtained in all the single insertion lines, but also in most of the lines with multiple insertions. Confirming the expected activities of the *AtSFH1* and *AtSFH3* promoters, examination under fluorescence microscopy revealed strongly fluorescing roots in the SFH1/DRTS2 lines and a strong fluorescence of the pollen grains in the anthers of the SFH3/DRTS3 lines (S3 Fig). On the contrary, fluorescence analyses of the SFH7/DRTS1 lines could not reveal any clear activity of the *AtSFH7* promoter. However, as suggested by microarrays data, the *AtSFH7* promoter may be able to drive weak expression in leaves where the interference caused by the presence of high amounts of chlorophyll could prevent the detection of eqFP611 fluorescence.

Concerning the histochemical GUS analyses of the transgenic lines, the consistent patterns of GUS staining observed with each construct revealed remarkable differences in the activity of the three *AtDRTS* promoters. Surprisingly, although the qRT-PCR analyses indicated the expression of *AtDRTS1* in both the root and shoot apices, GUS activity in all the SFH7/DRTS1 lines was uncertain in the shoot apex, due to strong vascular staining, and was not detectable in any of the root tips (Fig 3C, inset C1). This unexpected result suggested that regions required for the correct promoter activity in the apical meristems could be missing in the SFH7/DRTS1 construct. Because all the intergenic region upstream of *AtDRTS1* was included in the construct, we asked whether intragenic regions could be involved in the regulation of the *AtDRTS1* promoter. In this respect, several studies have previously reported that some of the introns of various genes can exert a strong influence on expression, an effect known as intron mediated enhancement (IME) [33]. In the majority of the cases this effect has been associated to the first intron, which is usually located in the 5’ untranslated region of the gene and close to the start of transcription, but examples of the influence of additional introns, even if located in the coding regions, have also been described. A software program, called IMEter, which is able to score the probability of introns to act as IME elements, has been developed [34] and is testable online at the web site http://korflab.ucdavis.edu/cgi-bin/IMEter_2014/web-imeter2.1.pl. As described in Fig 3B, according to the IMEter analysis of *AtDRTS1* both the first intron, located in the 5’ UTR and included in the SFH7/DRTS1 construct, as well as the second intron, located 420 bp downstream of the ATG codon and past the middle of the DHFR coding region, show remarkably high scores. Also in the case of *AtDRTS2* the IMEter analysis revealed high scores for the first and second intron, which are both included in the promoter construct (Fig 3A), whereas in the case of *AtDRTS3* a high score was observed for the first intron, which is located downstream to the ATG and is absent in the promoter construct. Considering the lack of GUS activity in root apices of the SFH7/DRTS1 transformants and the high IMEter score of the second intron of *AtDRTS1*, an additional promoter construct was prepared that extends to the beginning of the third exon of the gene. In this construct, called SFH7/DRTS1i2, the sequence coding for a large portion the amino-terminal DHFR domain of AtDRTS1 is fused in frame with the GUS coding sequence. Remarkably, the transgenic plants transformed with this construct revealed a strong GUS activity in the root apices indicating that the *AtDRTS1* promoter can drive expression in the root apical meristems only when the intragenic region that includes the second intron of the gene is present downstream of the promoter (Fig 3D to 3H). Interestingly a partly similar situation has been described for the *CENH3* gene of Arabidopsis, an E2F-regulated gene whose expression in the root apical meristems, but not in the shoot meristem, requires an intragenic region that includes the second intron of the gene [35].

**Fig 4:**
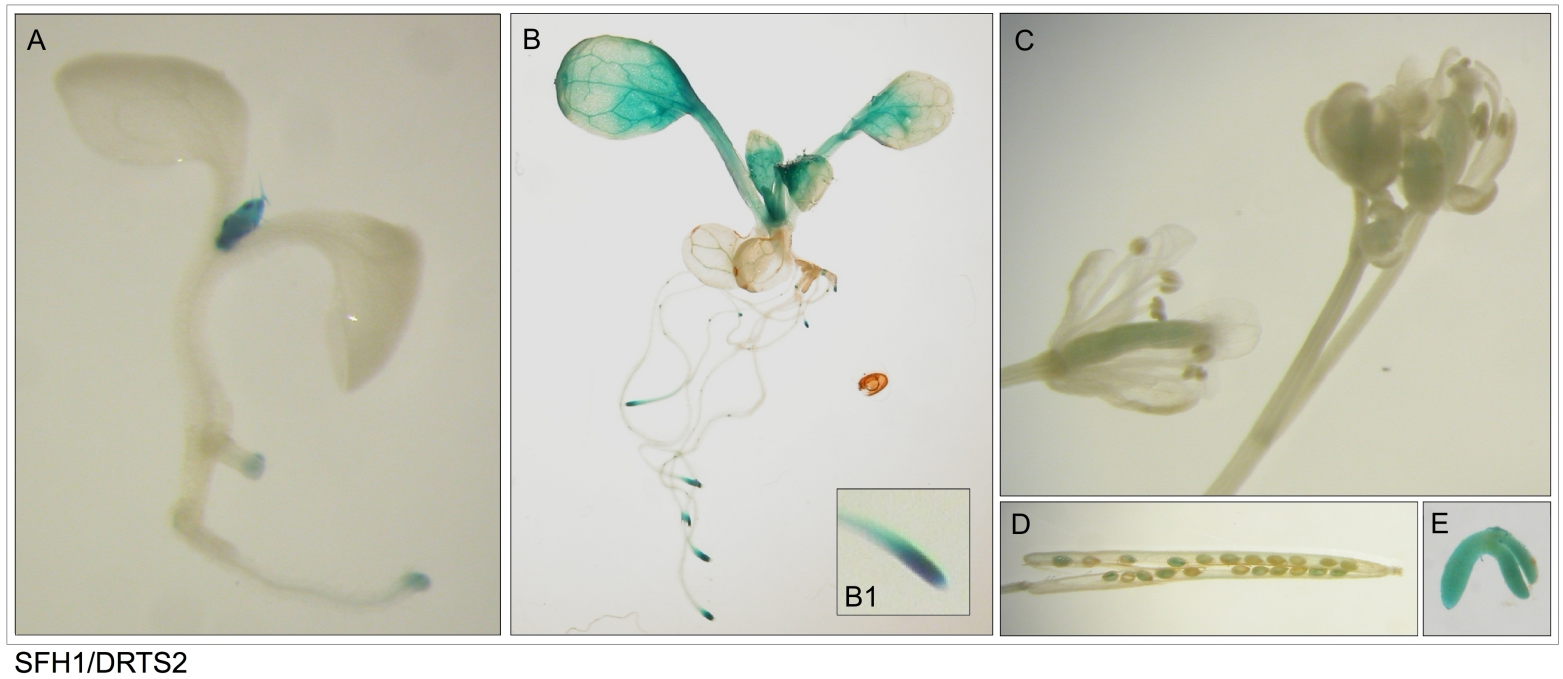
The *AtDRTS2* promoter is preferentially active in shoot and root apical meristems. (A to E) Localization of GUS activity in lines carrying the SFH1/DRTS2 construct. One-week-old (A) and two-week old (B) seedlings showing preferential activity of the *AtDRTS2* promoter in SAMs and RAMs (inset B1). GUS activity is detected weakly in carpels (C) and seeds (D) but is clearly seen in mature embryos (E).

**Fig 5:**
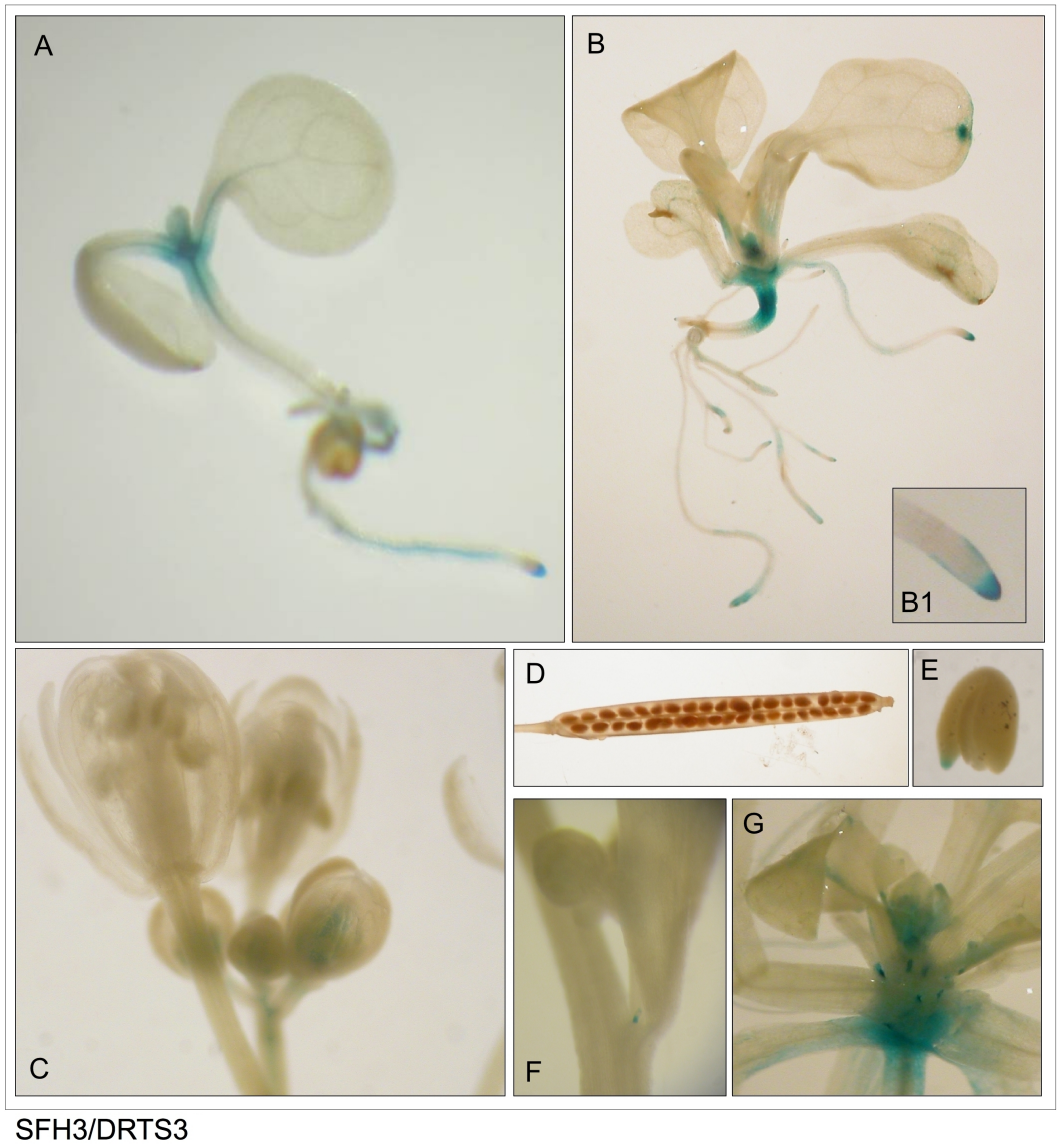
The *AtDRTS3* promoter is differentially active in the apical meristems. (A to G) Localization of GUS activity in lines carrying the SFH3/DRTS3 construct. One-week-old (A) and two-week old (B) seedlings showing preferential activity of the *AtDRTS3* promoter in SAMs, hydathodes and root caps (inset B1). Weak GUS activity is detected in small flower buds (C) and at the tip of the embryonic root of mature embryos (E), but is absent in the siliques (D). Localized activity of the *AtDRTS3* promoter is seen also at the insertion of lateral floral stems (F) and is strongly detected in stipules as well (G).

As shown in Figs 3–5 and described in Table 3, the patterns of activity of the three AtDRTS promoters are overlapping only partially and show distinctive features. As seen in the plants transformed with the SFH7/DRTS1i2 construct, the *AtDRTS1* promoter is able to drive expression of the *GUS* gene in both the shoot and root apical meristems, but is also broadly active in differentiated tissues of the roots, hypocotyls and cotyledons, which show particularly strong GUS staining of the vascular tissues (Fig 3D and 3E). The meristematic promoter activity is detectable also in lateral root primordia, whereas in mature leaves the *AtDRTS1* promoter apperas to be active in trichomes and in hydathodes (Fig 3E, inset E1). In mature flowers, the GUS staining can be detected in the style and ovary as well as in the vascular tissues of stamen filaments, whereas in developing flowers the promoter appears to be strongly active also in the stigmas (Fig 3F). Moreover, GUS activity is clearly detected also in maturing seeds and in embryos (Fig 3G and 3H). Thus, the *AtDRTS1* promoter appears to be highly active in meristematic tissues as well as in various differentiated tissues, in agreement with the pattern of expression detected by qRT-PCR. Also the promoter of *AtDRTS2* is strongly active in shoot and root apical meristems, but its activity in differentiated tissues is weak or undetectable in most tissues (Fig 4A and 4B). A gradient of GUS staining is evident at the base of developing leaves, where cell proliferation still occurs [36]. Particularly strong GUS staining is detected in lateral root primordia, which confirms that the *AtDRTS2* promoter is preferentially active in highly dividing cells. Very weak GUS staining can be detected in the ovary of flower buds (Fig 4C), whereas substantial promoter activity is detected in maturing seeds and in developing embryos (Fig 4D and 4E). Concerning the *AtDRTS3* promoter, the analysis of the SFH3/DRTS3 transformants revealed a clear GUS staining of the shoot apical meristems but lack of promoter activity in the apical meristems of the roots (Fig 5A and 5B). However, confirming the microarray and qRT-PCR data, a remarkably strong activity of the *AtDRTS3* promoter is found in the root cap (Fig 5B, inset B1) and weaker GUS staining is seen in the root vasculature as well. Surprisingly, the root cap-specific activity of the *AtDRTS3* promoter is already seen in developing embryos, which show localized GUS staining at the tip of the embryonic root (Fig 5E). Weak GUS staining can be seen also in the ovary in developing flower buds (Fig 5C), but staining of maturing seeds is not detectable in the siliques (Fig 5D). Moreover, in pSFH3/pDRTS3 plants the *AtDRTS3* promoter clearly active also in the hydatodes and in the stipules at the base of rosetta leaves (Fig 5B and 5G). Interestingly, in mature plants a localized activity of the *AtDRTS3* promoter can be detected in some cells at the branching point of the inflorescence stems (Fig 5F). In summary, the activities of the *AtDRTS* promoters observed in the transgenic Arabidopsis plants confirm and expand the data obtained by microarray and qRT-PCR analyses, revealing overlapping as well as specific patterns of expression of the *AtDRTS* genes. As described in Table 3, all three *AtDRTSs* are highly expressed in the SAM, but only *AtDRTS1* and *AtDRTS2* appear to be expressed in the RAM. Conversely, *AtDRTS3* is strongly expressed in the root cap, where expression of *AtDRTS1* and *AtDRTS2* does not occur. Moreover, *AtDRTS1* is broadly expressed also in cotyledons, leaves and in vascular bundles, whereas expression of *AtDRTS2* is mostly confined to the apical meristems and at the base of developing leaves, where cell proliferation occurs. *AtDRTS1* and *AtDRTS3* are also expressed in hydathodes but *AtDRTS3* is the only gene that is expressed strongly in the stipules.

**Table 3:** Patterns of GUS staining in the Arabidopsis lines transformed with the AtDRTS promoter constructs.

	DRTS1i2	DRTS2	DRTS3
RAM	++++	++++	-
SAM	+++	+++	+++
vascular bundles	+++	-	+
cotyledons	++	-	-
leaves	++	+	-
hydathodes-	+++	-	++
trichomes	++	-	-
stipules	-	-	+++
roots	++	-	+
Root caps	-	-	+++
pistils	++	+	+
stamens	+ (filaments)	-	-
siliques	++	+	-
embryos	++ (general)	++ (general)	+ (root tip only)

The analysis was performed on T2 progenies of all the single insert lines. Relative intensity of GUS staining is indicated as ++++, very strong; +++, strong; ++, moderate; +, weak; -, no staining.

### In silico analyses of the *AtDRTS* promoters reveal distinctive promoter architectures

The *AtDRTS* promoters, although with variable strength, appear to be all active in the shoot apical meristem and common regulatory circuits could be involved in their control in this specific context. However, the different patterns of expression observed in the root apical meristems and in other plant organs suggest also a distinctive regulation of the *AtDRTS* promoters. To verify the presence of common as well as specific regulatory elements in the *AtDRTS* promoters, *in silico* analyses were performed searching against the PLACE (http://www.dna.affrc.go.jp/PLACE/), JASPAR (http://jaspar.genereg.net/) and PlantPAN (http://plantpan2.itps.ncku.edu.tw/) databases, as well as using the RSAT (Regulatory Sequence Analysis Tools) (http://www.rsat.eu/) web platform. Because the 5’UTR of many genes were shown to contain functional *cis*-acting elements, the analyses were carried out including all the DNA sequences upstream of the ATG start codons. Moreover, the intergenic region upstream of the *AtDRTS* genes contains also the promoters of *AtSFH* genes and only 1311 bp separate the coding regions of *AtSFH1* and *AtDRTS2*, whereas the intergenic region upstream of the *AtDRTS1* ATG start codon is 1638 bp long and the *AtSFH3* and *AtDRTS3* ATGs are separated by 3471 bp. Considering the presence of the two promoters in the intergenic region, it is not possible to exclude that *cis*-acting elements that are involved in the regulation of the *AtSFH* genes could be influencing also the activity of the *AtDRTS* promoters. Thus, the promoter analyses were performed on the entire intergenic regions separating the *AtSFHs* and *AtDRTSs* ATG start codons, although we can assume that the *AtDRTS* genes are more likely to be regulated by putative *cis*-elements that are closer to their coding region than to the *AtSFH* genes. Dismissing very widespread short and low complexity elements, these analyses *in silico* allowed the identification of a moltitude of putative regulatory elements. Although only few of them are likely to be functional, their overall distribution suggests distinctive features of the three *AtDRTS* promoters. Altogether, 93 different putative regulatory sequences, varying in number and location, could be identified in at least one of the three intergenic regions (S3 Table). Interestingly, only 17 of these putative *cis*-acting elements are found in all three intergenic regions and only 9 of them are also invariably located, in one or more copies, at positions that could favour their involvement in the regulation of the *AtDRTS* genes. On the contrary, almost half of the remaining putative elements (44) are found, in single or multiple copies, in only one of the three intergenic regions. In this respect, 16 different *cis*-acting elements are found specifically in the region upstream of *AtDRTS1*, with 11 of them closer to the *AtDRTS1* coding region, and 5 elements are found only in the *AtSFH1/AtDRTS2* intergenic region, with 3 of them closer to *AtDRTS2*, whereas as many as 23 distintive putative regulatory sequences are found specifically in the *AtSFH3/AtDRTS3* intergenic region, 9 of which are also located at positions closer to *AtDRTS3* than to *AtSFH3* (S3 Table). In addition, considering that an intragenic region containing the second intron of *AtDRTS1* appears to be required for the activity of the *AtDRTS1* promoter in the root apical meristem, *in silico* analyses were performed to verify the presence of putative *cis*-acting elements also in this portion of the gene. In this respect, 11 putative regulatory sites are found in the second intron of *AtDRTS1*, three of which are not found in the intergenic region upstream of the coding region (S4 Table). Overall, the diversity of the putative regulatory elements that are found upstream and close to the *AtDRTS* genes, as well as the requirement of intragenic regions for full activity of the *AtDRTS1* promoter in root apical meristems, suggest very different architectures of the three *AtDRTS* promoters, that are likely to be controlled by distinctive transcriptional circuits.

**Fig 6:**
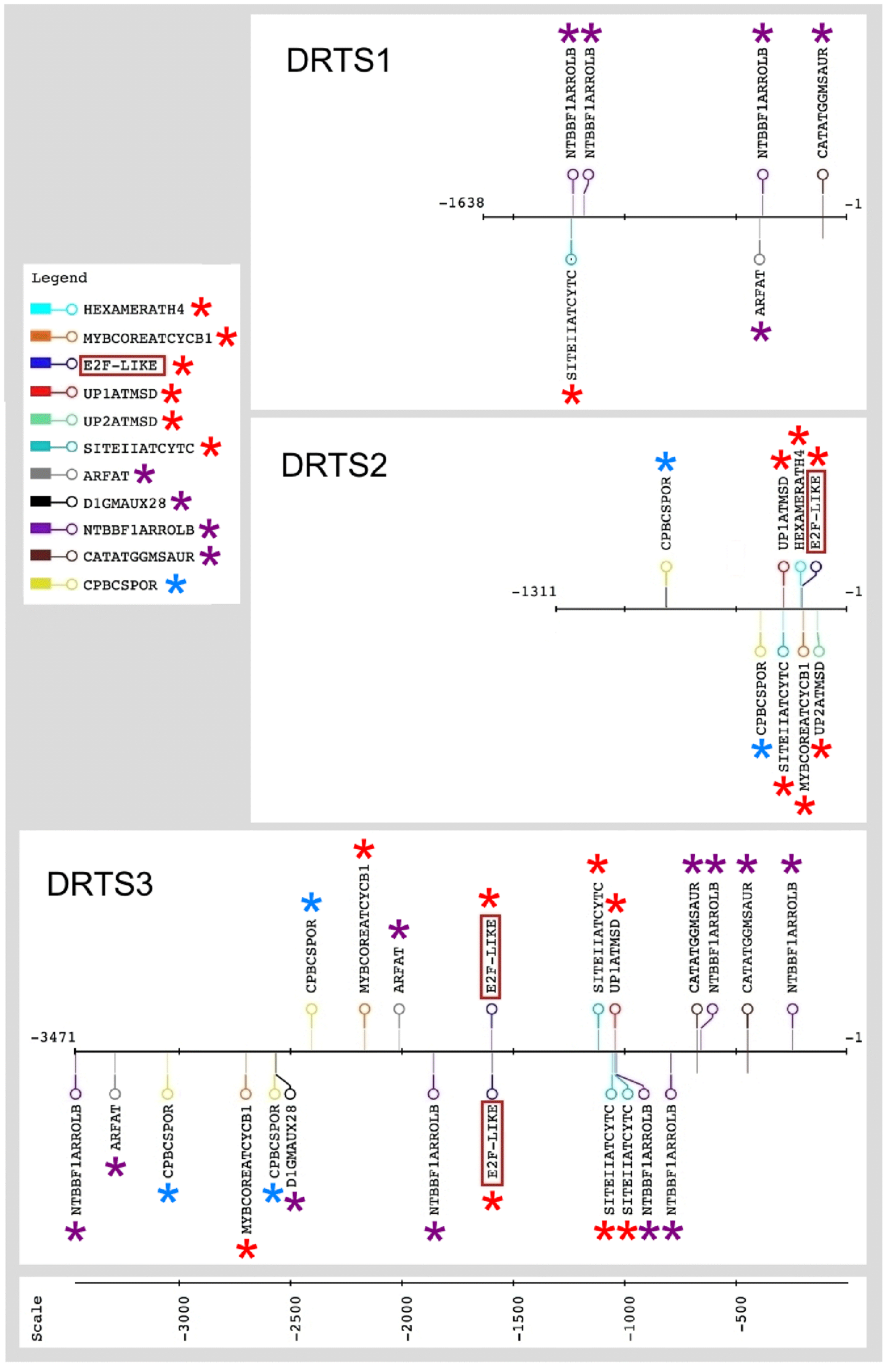
Putative *cis*-acting elements associated to cell proliferation or auxin and cytokinin response are differentially located upstream of the *AtDRTS* coding regions. Map of the relevant *cis*-acting elements identified in the intergenic regions separating the diverging *AtDRTS* and *AtSFH* coding sequences. Proliferation-related elements are marked with red asterisks whereas auxin-related sites are indicated with purple asterisks and the cytokinin-related CPBCSPOR sites are marked with light blue asterisks. The E2F sites found upstream of *AtDRTS2* and *AtDRTS3* are indicated with red boxes. The map was created using the drawing tool of the RSAT (Regulatory Sequence Analysis Tools) platform (http://www.rsat.eu/).

In particular, because DRTS activity is crucial for DNA synthesis and cell proliferation and all the *AtDRTS* promoters are able to drive expression in some meristematic tissues, we focused our attention on the presence of regulatory elements that have been reported to be involved in the control of gene expression in proliferating cells. Moreover, the balance of auxins and cytokinins plays important roles in the control of cell proliferation and *cis*-acting elements linked to auxin and cytokinin regulation of gene expression were also taken in consideration. The presence and the location of these putative regulatory sites in the intergenic regions upstream of the *AtDRTSs* is described in Fig 6, that highlights their distinctive distribution in the *AtDRTS* promoters. Most remarkably, various cell proliferation-related putative elements are found upstream of both the *AtDRTS2* and *AtDRTS3* coding regions, whereas only one putative site is found in the intergenic region upstream of *AtDRTS1*, but very close to the *AtSFH7* gene. Moreover, auxin-related elements are relatively abundant upstream of both *AtDRTS1* and *AtDRTS3* but are not detectable in the *AtDRTS2* promoter. Finally, CPBCSPOR, the only cytokinin-related regulatory element identified in this analysis, is not found in the *AtDRTS1* promoter but is detected twice upstream of AtDRTS2 and three times, although closer to *AtSFH3*, in the intergenic region upstream of *AtDRTS3*.

Because E2F transcription factors have been reported to regulate genes involved in DNA synthesis and cell proliferation in both plants and animals [37], the presence of putative E2F binding sites was investigated in detail. The E2F factors are known to bind specifically a consensus sequence TTTSSCGSS (where S can be C or G) and an E2FAT *cis*-element (TYTCCCGCC) has been reported in the promoters of many potential plant E2F target genes [38]. One copy of this element is actually found 199 nucleotides upstream of the *AtDRTS2* coding region but is not found upstream of the *AtDRTS1* and *AtDRTS3* coding regions. Nevertheless, recent studies based on chromatin immunoprecipitation ChIP-exo and ChIP-seq experiments have revealed that a shorter consensus element (TCCCGCC) is recognized *in vivo* by E2F factors [39,40]. A search for this sequence in the intergenic regions upstream of the *AtDRTSs* revealed the presence of a putative E2F binding site also 1591 nucleotides upstream of the ATG start codon of *AtDRTS3* but not in the promoter region of *AtDRTS1*. Remarkably, also using less stringent criteria to detect E2F-like elements (TSSCGSS) no additional putative E2F sites could be found in any of the intergenic regions upstream of the *AtDRTS* genes. Interestingly, the E2F-like element upstream of *AtDRTS3* is located in the middle of a transposon-like element and a recent study has reported that E2F sites are relatively common in plant transposable elements [41].

Most remarkably, concerning other *cis*–acting elements known to be particularly relevant for the regulation of genes expressed in proliferating cells, two of them are found only upstream and close to the *AtDRTS2* coding region. The first element is UP2ATMSD (AAACCCTA), which corresponds to the UP2 motif shown to be over-represented in the promoters of several genes that are up-regulated after main stem decapitation in Arabidopsis [42]. This putative *cis*-acting element is located at position -130 with respect to the ATG codon of *AtDRTS2*, next to the splice donor site in the first intron of the gene. The second element is HEXAMERATH4 (CCGTCG), the hexamer motif of Arabidopsis histone H4 promoter [43], that is located 208 nucleotides upstream of the *AtDRTS2* coding region. Other *cis*-acting elements linked to cell proliferation that are found upstream of some of the *AtDRTS* genes include UP1ATMSD (GGCCCAWWW), another motif enriched in the promoter of Arabidopsis genes up-regulated after main stem decapitation [42]. This site contains the SORLIP2AT motif (GGGCC), an element over-represented in light-induced promoters of Arabidopsis, and overlaps with the SITEIIATCYTC element (TGGGCY), a site involved in the regulation of the Arabidopsis *Cytc-1* promoter that is strongly active in root and shoot meristems [44]. Combined UP1ATMSD/SITEIIATCYTC elements are located upstream of both *AtDRTS2* and *AtDRTS3*, at positions that are much closer to the *AtDRTS* sequences than to the *AtSFH* genes and could favour their regulation of *AtDRTS2* and *AtDRTS3*, whereas two SITEIIATCYTC elements are found 1054 and 1116 bp upstream of the *AtDRTS3* coding region. Conversely, the intergenic region upstream of *AtDRTS1* does not contain any UP1ATMSD elements and contains only one SITEIIATCYTC element whose proximity to the *AtSFH7* coding region makes less likely its involvement in the control of *AtDRTS1* expression. Finally, a MYBCOREATCYCB1 site (AACGG), known to control the M-phase-specific expression of the Arabidopsis *cyclin B1*:*1* gene [45], is found at position -196 in the *AtDRTS2* gene and is also seen twice, although closer to the *AtSFH3* gene, in the intergenic region upstream of *AtDRTS3*, but is not detectable upstream of *AtDRTS1* (Fig 6). Based on the distinctive distribution of these putative regulatory sites, the activity of the *AtDRTS* promoters in apical meristems could be regulated differently. This is also stressed by the fact that the intragenic sequence of *AtDRTS1* required for promoter activity in root apical meristems does not contain putative regulatory elements reported to be involved in gene expression in proliferating cells.

Concerning the putative auxin-related *cis*-acting elements that can be detected upstream of *AtDRTS1* and *AtDRTS3*, but not upstream of *AtDRTS2*, one NTBBF1ARROLB site (ACTTTA) at position -377, one ARFAT site (TGTCTC) at position -392 and one CATATGGMSAUR site (CATATG) at position -108 are found shortly upstream of *AtDRTS1*, whereas in the *AtDRTS3* promoter, four of the five NTBBF1ARROLB sites and two of the three CATATGGMSAUR sites are closer to the *AtDRTS* gene and could be influencing its expression. As already mentioned and described in Fig 6, the CPBCSPOR element (TATTAG), corresponding to a sequence critical for the cytokinin-dependent binding of a nuclear protein to the *CsPOR* promoter of cucumber [46], is absent upstream of *AtDRTS1* but is found twice upstream of *AtDRTS2*, with one site at position -388 that could favour the control of *AtDRTS2*expression. Three CPBCSPOR sites are also found upstream of *AtDRTS3*, but their location very close to the *AtSFH3* coding region does not favour their involvement in *AtDRTS3* regulation.

### The *AtDRTS2* and *AtDRTS3* promoters are downregulated through E2F cis-acting elements

**Fig 7:**
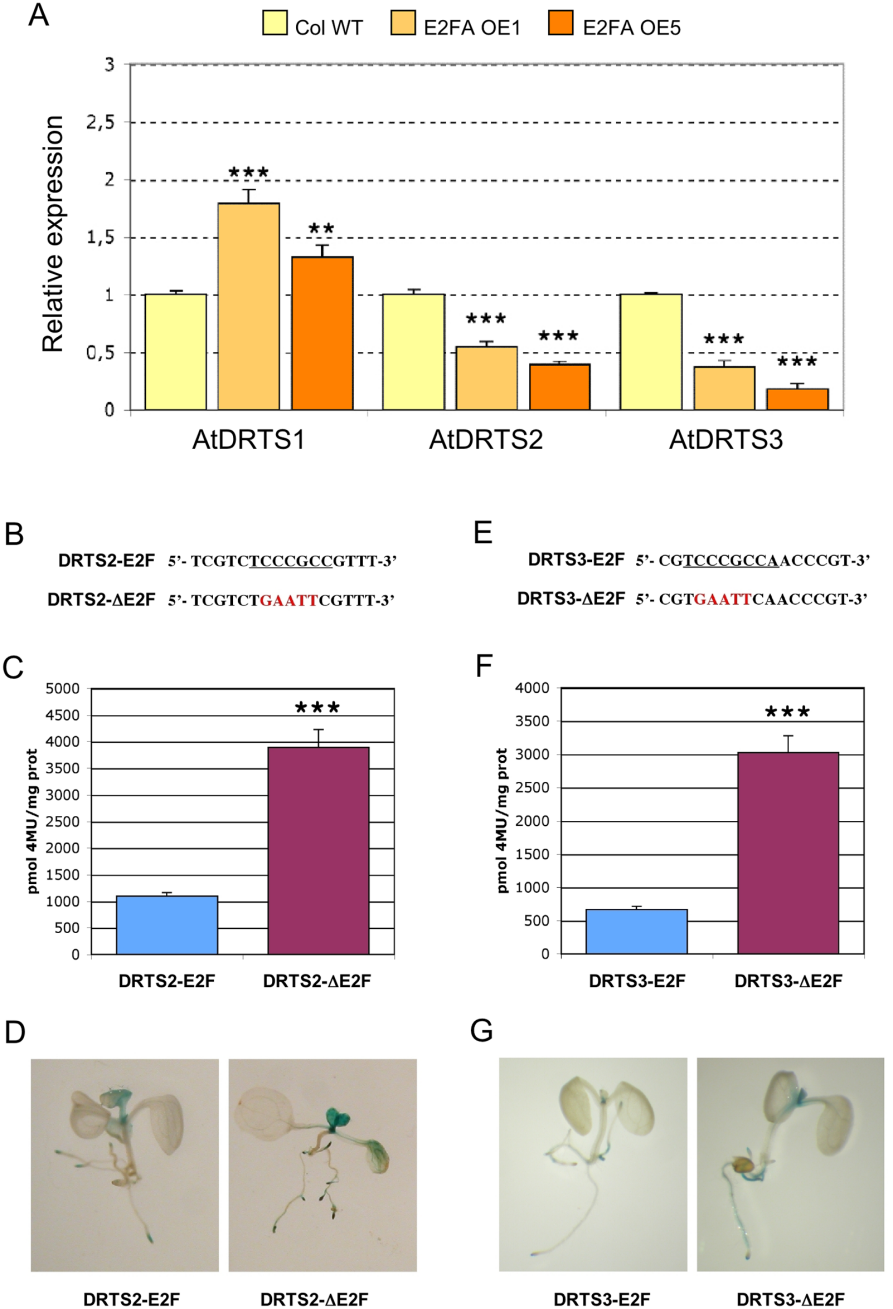
*AtDRTS2* and *AtDRTS3* are downregulated by E2F transcription factors. (A) *AtDRTS2* and *AtDRTS3* are downregulated in transgenic lines overexpressing the AtE2Fa factor whereas *AtDRTS1* expression increases. The qRT-PCR analyses were repeated three times using independent biological replicates and quantification was normalized to 18S RNA levels. The level of expression of the *AtDRTSs* in two E2Fa^OE^ lines compared to untransformed control is reported. The bars show standard errors. ** p<0.01, ***p<0.001. (B) to (G) The E2F-like sites in the *AtDRTS2* and *AtDRTS3* promoters exert repressive roles. Mutation of the E2F sites located upstream of *AtDRTS2* (A) and *AtDRTS3* (D) increases the strength of both promoters (B and E) without altering their spatial pattern of activity (C and F). Fluorimetric analysis of GUS activity in Arabidopsis plants harbouring the DRTS2 and DRTS2ΔE2F promoter constructs (B) or the DRTS3 and DRTS3ΔE2F promoter constructs (E) was carried out in triplicate on extracts from pooled two-week-old seedlings of all the single insertion transgenic lines. The bars show standard errors. ***p<0.001.

An E2F-dependent regulation of promoter activity has been reported for several genes involved in DNA synthesis and cell cycle progression in both plants and animals and the identification of E2F-like elements in the *AtDRTS2* and *AtDRTS3* promoters suggests that also these *DRTS* genes could be directly regulated by E2F factors. The family of these transcription factors includes typical E2Fs, which can act as activators or repressors depending on the cellular and promoter context, as well as atypical E2Fs, also called DELs, which are unable to activate gene expression and are believed to repress gene expression by competing with the activating E2Fs [47]. The typical E2Fs can bind DNA as heterodimers with DP partners, related proteins that provide a similar DNA binding domain, whereas the atypical E2Fs possess two DNA binding domains that allow DNA binding without the need of interactions with DP proteins. Arabidopsis possesses three typical E2F (AtE2Fa, AtE2Fb, AtE2Fc), three atypical E2Fs (AtE2Fd/DEL2, AtE2Fe/DEL1, AtE2Ff/DEL3) and two DPs (AtDPa, AtDPb) [48]. Interestingly, according to microarray analyses reported in the Genevestigator platform (https://www.genevestigator.ethz.ch/gv/index.jsp), overexpression of the AtE2Fa/AtDPa complex in transgenic plants leads to a slight increase in the expression of *AtDRTS1* and/or *AtDRTS2*, which correspond to the same probe set in the ATH1 microarray, whereas lower expression was detected for *AtDRTS3*. To better assess the influence of E2F factors on *AtDRTS* gene expression, Arabidopsis plants overexpressing AtE2Fa were produced and the expression of the three *AtDRTS* genes was analysed by qRT-PCR in two homozygous lines showing strong overexpression of the *AtE2Fa* transcripts (S5 Table). These lines, in agreement with previous reports concerning the overexpression of AtE2Fa or AtE2Fb [49,50], display a significant increase in cotyledonary epidermal cell number compared to wild type plants (S5 Table). Interestingly, increased expression of *AtDRTS1* can be detected in both the AtE2Fa^OE^ lines, whereas the expression of *AtDRTS2* and *AtDRTS3* clearly diminished (Fig 7A). These results suggest an E2F-dependent repression of the *AtDRTS2* and *AtDRTS3* promoters, which could be direct targets of E2F factors, and a positive influence of AtE2Fa overexpression on the expression of *AtDRTS1*, which is not necessarily reflecting a direct regulation but could be linked to the increased cell proliferation observed in the AtE2Fa^OE^ lines.

To confirm a direct repressive role of E2F factors in the regulation of *AtDRTS2* and *AtDRTS3*, critical mutations that are known to abolish E2F binding [48,51] were introduced in the E2F-like elements identified in the two promoters and the activity of the resulting constructs was investigated in transgenic plants. The sequence of the E2F-like site in the *AtDRTS2* promoter was changed from TCTCCCGCC into TCTGAATTC, whereas the E2F-like site in the *AtDRTS3* promoter was changed from CGTCCCGCC into CGTGAATTC (Fig 7B and 7E). The resulting SFH1/DRTS2ΔE2F and SFH3/DRTS3ΔE2F constructs were stably introduced in transgenic Arabidopsis plants and histochemical analyses performed on the T2 progeny revealed spatial patterns of GUS activity that are highly similar to those observed with the wild type promoters but the intensity of the GUS staining increased overall (Fig 7D and 7G). To verify the strength of the mutated promoters compared to the wild type ones, extracts of hygromycin resistant T2 seedlings of all the available transgenic lines were assayed fluorimetrically to evaluate the GUS specific activities. As shown in S4 Fig, highly variable levels of GUS activity could be detected in the different lines, possibly influenced by post-transcriptional gene silencing linked to the insertion of multiple copies of the transgene at single or multiple sites [52]. Nevertheless, a general increase of activity was apparent in the lines transformed with the mutated promoter constructs compared to the wild type constructs (S4 Fig). To quantify the increased strength of the mutated promoters, additional fluorimetric analyses were performed in triplicate with extracts obtained pooling T2 seedlings of all the single insertion lines because their GUS activities appeared to be more uniform and transgenic lines containing single-copy T-DNAs have been previously shown to display uniform and comparable levels of GUS expression [53]. As described in Fig 7C and 7F, the mutation of the E2F-like site in the *AtDRTS2* and *AtDRTS3* promoters increased significantly the activity of both promoters compared to the wild type constructs. These results demonstrate that the E2F-like elements identified in the *AtDRTS2* and *AtDRTS3* promoters are functional and can exert a repressive role in the control of both promoters.

### The meristematic activity of the *AtDRTS1* and *AtDRTS2* promoters in germinating seeds is cell cycle-regulated

**Fig 8:**
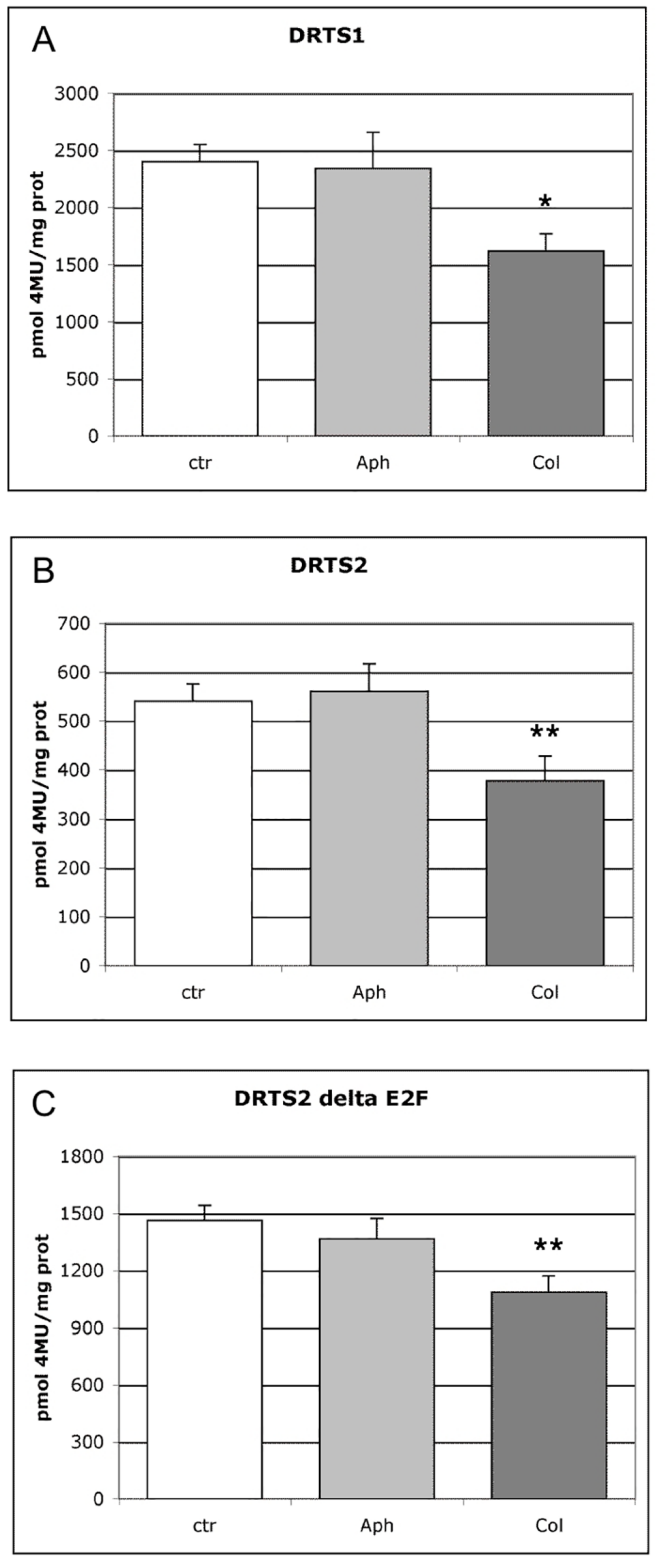
The activity of the *AtDRTS1* and *AtDRTS2* promoters is cell cycle regulated in germinating seeds. Fluorimetric analysis of GUS activity in transgenic lines harbouring the DRTS1 (A), DRTS2 (B) and DRTS2ΔE2F (C) promoter constructs was carried out on extracts obtained from germinating seeds incubated 72 h without (ctr) or with cell cycle inhibitors (Aph, Col). The results obtained with one representative line for each construct are presented. The analyses were carried out using three biological replicates. Treatment with colchicine lowers significantly the GUS activity of germinating seeds of each line. The bars show standard errors. *p<0.05, **p<0.01.

Considering the crucial role played by the DRTS enzymes in DNA synthesis, the expression of the *AtDRTS* genes in proliferating cells is expected to be preferentially linked to the G1/S phase of the cell cycle. To investigate the cell cycle-dependent regulation of *AtDRTS1* and *AtDRTS2*, which are both highly expressed in proliferating embryonic cells and in root apical meristems, experiments using cell cycle inhibitors were performed with germinating seeds characterized by synchronous cell cycle progression during the early stages of germination [54]. In dormant dry seeds most of the cells of the embryo are known to be blocked at the G1 phase. Upon seed imbibition, cells in the radicle progress into S phase and start the synthesis of DNA, which terminates approximately 42 hours after imbibition (HAI), when the radicle starts to protrude. Then the cells pass into the G2 phase, which is followed shortly by the M phase that occurs approximately 48 HAI and leads to the beginning of a new cell cycle in the cells obtained after the first division. As shown also by Varadarajan et al. [55], imbibition and germination of the seeds in the presence of aphidicolin appears to block the cells in S phase, while the germination in the presence of colchicine allows the completion of the first S phase and blocks the cells at the M phase. To analyse the activity of the *AtDRTS1* and *AtDRTS2* promoters during seed germination, seeds of representative SFH7/DRTS1i2 and SFH1/DRTS2 lines that show a clear GUS activity from the beginning of germination, were imbibed for 74 hours in the dark at room temperature with or without cell cycle inhibitors. In the absence of inhibitors, this length of time would allow the meristematic cells to complete two divisions. The extracts from the germinated seeds were then analyses fuorimetrically to quantify the level of GUS activity. As shown in Fig 8A and 8B, germination in the presence of colchicine decreased significantly the GUS activity in the seeds of the transgenic lines, whereas incubation with aphidicolin did not alter significantly the level of GUS activity compared to control seeds germinated without the inhibitors. Although it is known that the GUS protein is particularly stable and can persist during cell cycle progression in proliferating cells [56], these results suggest that the activity of the *AtDRTS1* and *AtDRTS2* promoters in germinating seeds is partly cell cycle-regulated, being high in G1/S but low or absent in G2/M. In this respect, we asked whether the negative control exerted by the E2F *cis* elements on the activity of the *AtDRTS2* promoter, which is strongly active only in the apical meristems, could represent a general repression or could be a way to downregulate specifically the expression of *AtDRTS2* in the G2 and M phase of the cell cycle. The increased meristematic strength of the promoter mutated at the E2F-like site could then result from an extension of its activity throughout the entire cell cycle. As shown in Fig 8C, performing the analysis on a representative line harbouring the SFH1/DRTS2ΔE2F construct revealed that the inactivation of the E2F-like element does not affect the response to cell cycle inhibitors. It appears therefore that the E2F site in the *AtDRTS2* promoter can play a general repressive function and is not involved in the cell cycle dependent regulation of *AtDRTS2*.

## Discussion

In this study we describe a molecular characterization of the three members of the *DRTS* gene family of *Arabidopsis thaliana*, revealing the existence of remarkable isoforms and of distinctive promoter features reflecting differential patterns of expression. The *DRTS* genes are peculiar to plants and protists and code for bifunctional proteins characterized by the union in a single molecule of the domains specifying two enzymatic activities, dihydrofolate reductase (DHFR) and thymidylate synthase (TS), which in animals, fungi and bacteria are encoded by separate genes. DHFR catalyses the last reaction in the synthesis of tetrahydrofolate (THF), whereas TS uses N5,N10-methylene THF to reduce and methylate deoxyuridine monophosphate (dUMP) to dTMP, yielding 7,8-dihydrofolate (DHF) as a secondary product. Because DHFR activity is needed to recycle the resulting DHF, TS relies on DHFR activity and the presence of both enzymes in the same polypeptide, known as metabolic channelling, clearly increases the efficiency of thymidylate synthesis.

### Different *AtDRTS* isoforms are expressed and some are expected to code for monofunctional DHFR enzymes

All three *DRTS* genes of Arabidopsis are downstream to divergently oriented members of the *AtSFH* gene family and could derive from successive genome duplications that occurred during Brassicaceae evolution. The AtDRTS1 and AtDRTS2 proteins are more similar to each other than to AtDRTS3 and form a clade together with a group of other Brassicaceae DRTSs. On the contrary, AtDRTS3 groups with a subset of DRTSs conserved also in other eudicots. It appears, therefore, that *AtDRTS1* and *AtDRTS2* could derive from a recent duplication event that occurred after the separation of Brassicaceae from other plant families and before the divergence of the Arabidopsis and Brassica lineages [57]. In all the plant and protist DRTS proteins the amino-terminal DHFR domain and the carboxy-terminal TS domain are separated by a linker region whose variable structure reflects evolutionary changes and has been used as marker for phylogenetic classification [58]. Although length and sequence of the linker region have been shown to be critical for TS activity and domain-domain interaction of the bifunctional enzyme [19], the two enzymatic activities appear to be largely autonomous and inhibition of each one with specific drugs does not affect the other activity [59]. In this respect, because the synthesis of THF is not needed only for TS activity but is necessary for a myriad of other metabolic pathways, it is not surprising that DHFR activity in plant cells has been reported to be at least 20 to 30 folds higher than TS activity. Moreover, the domain responsible for TS activity appears to be much more sensitive to protease action than the DHFR domain [59]. Interestingly, earlier studies have suggested the existence in plants of monofunctional DHFRs, associated with TS in a large multimeric enzyme complex [60]. Although monofunctional DHFRs could derive from partial degradation of the TS moiety, our analysis of the Arabidopsis *DRTS* gene family reveals that alternatively spliced isoforms of *AtDRTS1* and *AtDRTS3* are potentially coding for truncated proteins that are expected to possess only DHFR activity. The differential splicing of *AtDRTS3* transcripts is likely associated to the presence of a transposon-like element in the fourth intron of the gene, causing a termination of the primary transcripts before reaching the regular 3' splicing acceptor site of the intron. Alternative splicing has been detected also for the *AtDRTS2* transcripts and is expected to results in the use of two different ATG codons, giving rise to protein isoforms possessing different amino-terminal regions. According to various targeting predictions, these AtDRTS2 isoforms could be localized to different sub-cellular compartments and the larger one is mostly expected to be targeted to mitochondria and/or plastids, whereas the smaller one is mainly predicted to be cytosolic. A similar scenario has been reported also for a carrot *DRTS* gene showing alternative transcription starts that give rise to two isoforms, one of which possesses a N-terminal region with the features of a transit peptide that could target the protein to the plastids [61]. Mitochondrial localization of plant DRTSs is very likely because a huge pool of THF is needed for the photorespiratory process in leaf mitochondria of C3 plants and folate and thymidylate synthesis in plants have been shown to occur predominantly in mitochondria [59]. Nevertheless, compartmentalization of plant DRTSs is still an open question and, as predicted for the smaller AtDRTS2 isoform, a cytosolic localization is mostly proposed also for the AtDRTS1 and AtDRTS3 proteins. Thus, it is possible that in particular cellular or developmental contexts some of the AtDRTSs could be localized, to various extents, not only in mitochondria but also in plastids and in the cytosol as well.

### The differential expression of the *AtDRTS* genes suggests alternative functions

Expression analyses, conducted by qRT-PCR and evaluating the activity of the *AtDRTS* promoters in transgenic plants, revealed that the *DRTS* genes of Arabidopsis are variously expressed in meristematic and differentiated cells. Moreover, the distinctive patterns of expression of the three *AtDRTS* genes in differentiated tissues suggest specific roles not necessarily linked to cell proliferation or endoreduplication. In this respect, *AtDRTS1* appears to be the most widely expressed gene and its promoter is strongly active in the vascular tissues, whereas *AtDRTS2* and *AtDRTS3* show narrower and more specific patterns of expression. The strong expression of AtDRTS1 in vascular tissues emphasizes the important roles played by folates in the synthesis of lignin and of other cell wall components [62]. Of all three genes, *AtDRTS2* is the only one that is predominantly expressed in meristematic tissues. Meristematic expression is clearly linked to the need of thymidylate for DNA synthesis in proliferating cells, whereas the expression in many differentiated cells could be associated to DNA endoreduplication or to the synthesis of the folate cofactors required for various biochemical reactions. With respect to the meristematic expression, all three genes appear to be expressed in shoot apices, with *AtDRTS1* and *AtDRTS2* showing considerably higher expression compared to *AtDRTS3*. Also developing ovaries, in which cell proliferation occurs, show weak activity of all three *AtDRTS* promoters, whereas the expression of the *AtDRTSs* in other tissues and organs appears to be shared by only two of the genes or is rather a prerogative of *AtDRTS1* or *AtDRTS3*. Interestingly, root apical meristems exhibit strong expression of both *AtDRTS1* and *AtDRTS2* but there is no evidence of the expression of *AtDRTS3*, which is the only *AtDRTS* gene that in the root apex is strongly expressed in the columella and in the lateral root cap. Thus, although strong meristematic expression of *AtDRTS1* and *AtDRTS2* is evident in both shoot and root apical meristems, meristematic expression of *AtDRTS3* appears to be restricted to the shoot apex only. Moreover, *AtDRTS1* and *AtDRTS2* are expressed widely in developing embryos, whereas the expression of *AtDRTS3* appears to be confined to a narrow region at the very tip of the embryonic root. These results suggest that *AtDRTS1* and *AtDRTS2* are consistently expressed in all the cells that undergo proliferation, whereas the expression of *AtDRTS3* in proliferating cells occurs but is restricted to particular developmental or spatial contexts. In addition, the effects of cell cycle inhibitors on the activity of the *AtDRTS1* and *AtDRTS2* promoters in germinating seeds revealed that both genes are cell cycle-regulated in root meristems, showing higher expression at the G1/S phase in accordance with their importance for DNA synthesis.

Although the activities of the *AtDRTS1* and *AtDRTS3* promoters are partially overlapping in hydathodes, the *AtDRTS1* and *AtDRTS3* promoters show specific patterns of activity in other parts of the plant. The promoter of *AtDRTS1* appears to be active in trichomes and in the developing stigmatic papillae of flower buds, but not in mature stigmas of open flowers. The expression of *AtDRTS1* in these cell types could be linked to endoreduplication, which has been shown to occur during both trichomes and stigmatic papillae development [63]. Conversely, the *AtDRTS3* promoter appears to be active in the stipules and in few cells at the adaxial base of inflorescence branches. This expression recalls the activity of two *PECTATE LYASE-LIKE (PLL)* promoters, *PLL15* and *PLL24*, that have been shown to drive *GUS* gene expression within a restricted region on the adaxial side of the base of pedicels and, as most other *PLL* promoters, are also active in the stipules of Arabidopsis plants [64] The function of stipules is still unclear but, together with hydathodes, they are believed to be primary sites for the synthesis of indole-3-acetic acid (IAA) associated with vascular differentiation and leaf morphogenesis [65]. The transcription of *AtDRTS1* and *AtDRTS3* genes at sites of IAA synthesis could reflect an auxin-dependent upregulation of their promoters, also supported by the presence of several auxin-responsive *cis*-acting elements. However, the expression of *AtDRTS* genes in stipules, hydathodes and root caps could also imply links between folates and IAA biosynthesis. Auxin distribution and signaling have been recently shown to be modulated by interactions between folate biosynthesis and Sucrose signaling [66]. Moreover, it is known that 5,10-CH2-THF is a methyl donor for the synthesis of CoA molecules that, in addition to their engagement in various metabolisms, are also necessary for the β-oxidation of the auxin precursor indole-3-butyric acid (IBA) [67]. Interestingly, expression in lateral root cap cells of the *IBR3* gene, encoding a protein involved in the conversion of IBA into IAA, has been shown to create a local auxin source that stimulates LR formation [68].

The strong and specific expression of *AtDRTS3* in the columella and lateral root cap could support also other functions. Root caps protect the RAM and play important roles in root growth allowing gravity perception. High metabolic activity is known to occur in root cap cells, which secrete mucilage to facilitate root penetration in the soil and release diverse secondary metabolites that influence rhizosphere microbiota composition [69]. Root border cells also produce antimicrobial phenolic compounds important in the defence against fungal pathogens and DHFR activity in root cap cells could be involved in the production of these defensive molecules. A recent study of Arabidopsis root exudates has revealed the release of a large array of compounds, including thymidine and degradation products of methionine-derived glucosinolates [70]. Moreover, DNA synthesis has been reported to occur in root cap border cells [71,72] and the release of large amounts of extracellular DNA (exDNA) by root tips has been shown to play important defensive roles [73] Together with secreted proteins, the released exDNA forms traps that are able to block pathogens and protect growing root tips from invasion. The defensive role of the exDNA is fully demonstrated by the loss of resistance upon treatments with DNaseI and by the capacity of bacterial strains to release nucleases to increase their virulence. Although the release of exDNA by root border cells has not been investigated in Arabidopsis, this feature has been described in different plant species and is likely to be widespread. Thus, it is possible that the strong expression of *AtDRTS3* in root caps is associated to the occurrence of this phenomenon also in Arabidopsis. Interestingly, important involvements of folate metabolism with plant defence have been already suggested. Folate content in rice seeds is associated with the induction of defence-related genes [74] and folic acid has been shown to induce local and systemic SA-mediated defence in Arabidopsis [75]. Moreover, in a study carried out in maize, two QTL that relate to brown plant-hopper (BPH) resistance have been shown to be associated with *ZmDRTS* genes [76]. Although redundancy of the *AtDRTS* genes could support vital functions related to general metabolism and cell proliferation, functional analyses of the individual *AtDRTS* genes will be useful to assess whether *AtDRTS1* or *AtDRTS3* can be involved in auxin distribution and signaling and to verify whether *AtDRTS3* can play important roles in plant defense from pathogens.

### Distinctive promoter architectures suggest that the *AtDRTS* genes are controlled by different regulatory circuits

According to the distinctive patterns of expression observed, the three *AtDRTS* genes appear to be differentially regulated to a large extent. In agreement with this finding, *in silico* analyses of the *AtDRTS* promoters revealed remarkably different distributions of several putative regulatory sites. Focusing our attention on *cis*-acting elements reported to be involved in the regulation of gene expression in proliferating cells and in response to auxin and cytokinin, we found clear differences among the *AtDRTS* promoters. Several proliferation-related elements are found upstream and closer to the *AtDRTS2* and *AtDRTS3* coding regions whereas the only putative site found upstream of *AtDRTS1* is much closer to the *AtSFH* gene than to the *AtDRTS* coding region and is less likely to be possibly involved in *AtDRTS1* regulation. Moreover, we discovered that the expression of *AtDRTS1* in root meristems, as described also for the *CENH3* gene of Arabidopsis [35], is strictly dependent on the presence of an intragenic region that includes the second intron of the gene, in which proliferation-related *cis*-acting elements are absent. The *AtDRTS2* promoter, which shows mostly meristematic activity, is particularly enriched in proliferation-related regulatory sites and contains six different putative *cis*-acting elements that are associated to expression in proliferating cell and are all grouped closely upstream of the ATG codon. In comparison, the *AtDRTS3* promoter contains four diverse proliferation-related regulatory elements that are more dispersed and distant form the *AtDRTS* coding region. Thus, although overlapping patterns of expression are seen in some meristematic tissues, the remarkably distinctive promoter architectures of the three *AtDRTS* genes suggest that their expression in proliferating cells could be controlled to a large extent by different regulatory circuits.

Considering the presence of auxin-responsive *cis*-acting elements, it is notable that various sites related to auxin regulation are found only in the *AtDRTS1* and *AtDRTS3* promoters, that are strongly active at sites of auxin production, but are absent in the *AtDRTS2* promoter. Conversely, two and three copies of a putative cytokinin-responsive element are seen upstream of *AtDRTS2* and *AtDRTS3*, respectively, but are absent in the *AtDRTS1* promoter. Nevertheless, additional studies will be needed to confirm the relevance of these putative regulatory sites and to verify whether the expression of *AtDRTS1* and *AtDRTS3* can be actually regulated by auxin whereas cytokinin could control *AtDRTS2* and *AtDRTS3* expression.

### The *AtDRTS2* and *AtDRTS3* promoters are partly regulated through E2F *cis*-acting elements

Among proliferation-related *cis*-acting elements, the E2F sites are believed to play particularly important roles and have been shown to to be required for meristematic expression of some plant genes [47]. However, functional analyses of the E2F-like sites in the *AtDRTS2* and *AtDRTS3* promoters suggest that both genes are negatively regulated by endogenous E2F factors. An E2F-dependent regulation of *DRTS* genes has never been described before and the control of mammalian *DHFR* and *TS* genes by E2F factors appears to be controversial because various studies have shown contrasting results and strong regulation occurs at the post-transcriptional level [77,78]. An E2F-mediated repression of *AtDRTS* genes was confirmed also in plants overexpressing AtE2Fa, in which *AtDRTS2* and *AtDRTS3* are downregulated whereas *AtDRTS1*, which lacks putative E2F sites, appears to be upregulated. *AtDRTS1* is not expected to be an E2F target but is strongly expressed in proliferating cells and its upregulation in AtE2Fa^OE^ plants could simply reflect the increased cell proliferation caused by AtE2Fa overexpression.

The activity of typical E2Fs is finely controlled by post-translational modifications and through their association with Retinoblastoma-Related (RBR) proteins. Free typical E2Fs can function as activators, but the interaction with RBRs confers them repressive roles [79]. Among the typical AtE2Fs, AtE2Fa and AtE2Fb have been proposed to act mainly as transcriptional activators that are able to upregulate the expression of several cell cycle genes. On the contrary, AtE2Fc, the third typical E2F of Arabidopsis, as well as the three atypical E2Fs, AtE2Fd to AtE2Ff, are believed to act mainly as repressors of E2F-regulated genes [47]. However, repressive roles of AtE2Fa and AtE2Fb have been also reported. In apical meristems, AtE2Fa was shown to be mostly associated with AtRBR1 to repress genes involved in endoreduplication and cell expansion, whereas AtE2Fb is believed to interact with RBR1 only in elongating and differentiating cell, repressing cell cycle genes in cells leaving the meristems [80]. The downregulation of *AtDRTS2* and *AtDRTS3* in AtE2Fa^OE^ plants could then be linked to the repressive role exerted by AtE2Fa on genes involved in DNA synthesis in proliferating cell. Nevertheless, the overexpression of AtE2Fa has been shown to upregulate AtRBR1 and remarkable interplays are known to occur among E2F genes, many of which appear to be E2F-regulated [50,81–83]). Thus, we cannot rule out the possibility that the downregulation of *AtDRTS2* and *AtDRTS3* in AtE2Fa^OE^ plants is not exerted directly by AtE2Fa, but could result from an upregulation of repressive E2Fs caused by AtE2Fa overexpression. In any case, our results clearly suggest that *AtDRTS2* and *AtDRTS3*, but not *AtDRTS1*, are negatively controlled by E2F factors. Remarkably, also the *AtCNH3* gene has been proposed to be downregulated by E2F factors in Arabidopsis protoplasts, even if its expression appeared to increase in plants overexpressing AtE2Fa or AtE2Fb [35]. Repressive E2F sites have been reported also for other plant promoters and in some cases have been associated to downregulation at specific phases of the cell cycle or to repression in mature organs [84,85]. Although the *AtDRTS2* promoter appears to be cell cycle-regulated in root meristems, with maximal activity at G1/S phase, the inactivation of its E2F site did not alter its pattern of activity during the cell cycle, suggesting that the E2F-dependent control of both *AtDRTS2* and *AtDRTS3* promoters allows a general downregulation of their activity in both meristematic and differentiated cells and is not linked to their regulation at specific phases of the cell cycle.

## Conclusions

With this study we discovered a complex differential regulation of the *AtDRTS* genes that confirms their expected involvement in cell proliferation and endoreduplication, but indicates also peculiar functions related to distinctive cellular activities. High expression at primary sites of auxin synthesis suggests links with auxin metabolism and/or functions, whereas strong vascular expression may be associated to the synthesis of lignin and of other components necessary for secondary wall formation. Moreover, a particularly strong root cap-specific expression of *AtDRTS3* could imply its involvement with root protection. The identification of differentially spliced transcripts coding for AtDRTS isoforms lacking most of the carboxy-terminal TS domain may account for the proposed existence in plant cells of monofunctional DHFR enzymes that could support the need of folates for pathways unrelated to DNA synthesis. Finally, evaluation of the expression of the *AtDRTS* genes in AtE2Fa^OE^ plants and functional analyses of the *AtDRTS* promoters suggest a negative regulation of *AtDRTS2* and *AtDRTS3* by E2F factors that appear to exert a general repression and are not involved in the cell cycle-dependent regulation of the *AtDRTS* genes. These results expand our knowledge on the function and control of plant *DRTS* genes and suggest that E2F activities are not determining the meristematic expression of the Arabidopsis *DRTS* genes.

## Supporting information

S1 Fig Evolutionary relationships of angiosperm DRTS proteins. The Phylogenetic Tree was created aligning the aminoacid sequences with Clustal Omega (http://www.ebi.ac.uk/Tools/msa/clustalo/). The branches including monocots, Fabaceae and brassicaceae are pointed out. The AtDRTSs are indicated with red boxes.
(TIF)

S2 Fig Spatial patterns of accumulation of the *AtDRTS1/2* and *AtDRTS3* transcripts according to microarray analyses. Data shown are reported at the Botany Array Resource (BAR) Browser (http://bar.utoronto.ca/).
(TIF)

S3 Fig Visualization of eqFP611 red fluorescence driven by the *AtSFH1* (A) and *AtSFH3* (B) promoters. The fluorescence was visualized *in vivo* with a Wild M10 stereomicroscope equipped with a Leica fluorescence module using a standard TRITC filter set.
(TIF)

S4 Fig Analysis of GUS activity in the T2 progeny of all the individual lines transformed with wild type or ΔE2F DRTS2 and DRTS3 promoter constructs. The extracts were prepared form two-week-old hygromycin resistant seedlings. The mean values of triplicate experiments are reported for each line.
(TIF)

S1 Table Primers used for PCR amplification and production of the SFH/DRTS promoter constructs. The restriction sites incorporated in the primers and used to clone the PCR fragments are underlined.
(DOC)

S2 Table Primers used for qPCR analysis. (DOC)

S3 Table Presence and location of *cis*-acting elements in the SFH/DRTS intergenic regions. The analyses were performed searching against the PLACE (http://www.dna.affrc.go.jp/PLACE/), PlantPAN (http://plantpan2.itps.ncku.edu.tw/) and JASPAR (http://jaspar.genereg.net/) databases. The distance from the *AtDRTSs* ATG codon is reported. Sites that are closer to the *AtDRTS* coding region than to *AtSFH* are indicated in red. *Cis* elements related to expression in proliferating cells, endosperm expression and hormone response are highlighted in different colors.
(DOC)

S4 Table Presence and location of *cis* elements in the intragenic 5’ region of *AtDRTS1*. The analyses were performed searching against the PLACE (http://www.dna.affrc.go.jp/PLACE/), PlantPAN (http://plantpan2.itps.ncku.edu.tw/) and JASPAR (http://jaspar.genereg.net/) databases. Position from ATG corresponds to the bp distance downstream from the ATG codon. Sites located within the second intron of the *AtDRTS1* gene are shown in red.
(DOC)

S5 Table Features of the two Arabidopsis lines overexpressing the AtE2Fa factor. The accumulation of the *AtE2Fa* mRNA in one-week-old seedlings was quantified by qPCR following the ΔΔ^Ct^ method using the18S RNA as a reference for normalization. The qPCR analysis was carried out using three biological replicates. The mean level of expression with the SE is reported. The phenotypic analysis of the cotyledons was carried out on 12-day-old plants using 8 to 12 samples. The size of the adaxial epidermal cells was calculated counting the number of cells contained in an area of 100,000 μm^2^. The total epidermal cell number was estimated dividing the cotyledon size by the cell size. The mean values with the SE are reported.
(DOC)
