# Supplementary figures and images for: Distinctive features and differential regulation of the DRTS genes of Arabidopsis thaliana
Source: PLoS One. 2017 Jun 8;12(6):e0179338. doi: 10.1371/journal.pone.0179338 (PMC5464667; doi:10.1371/journal.pone.0179338)

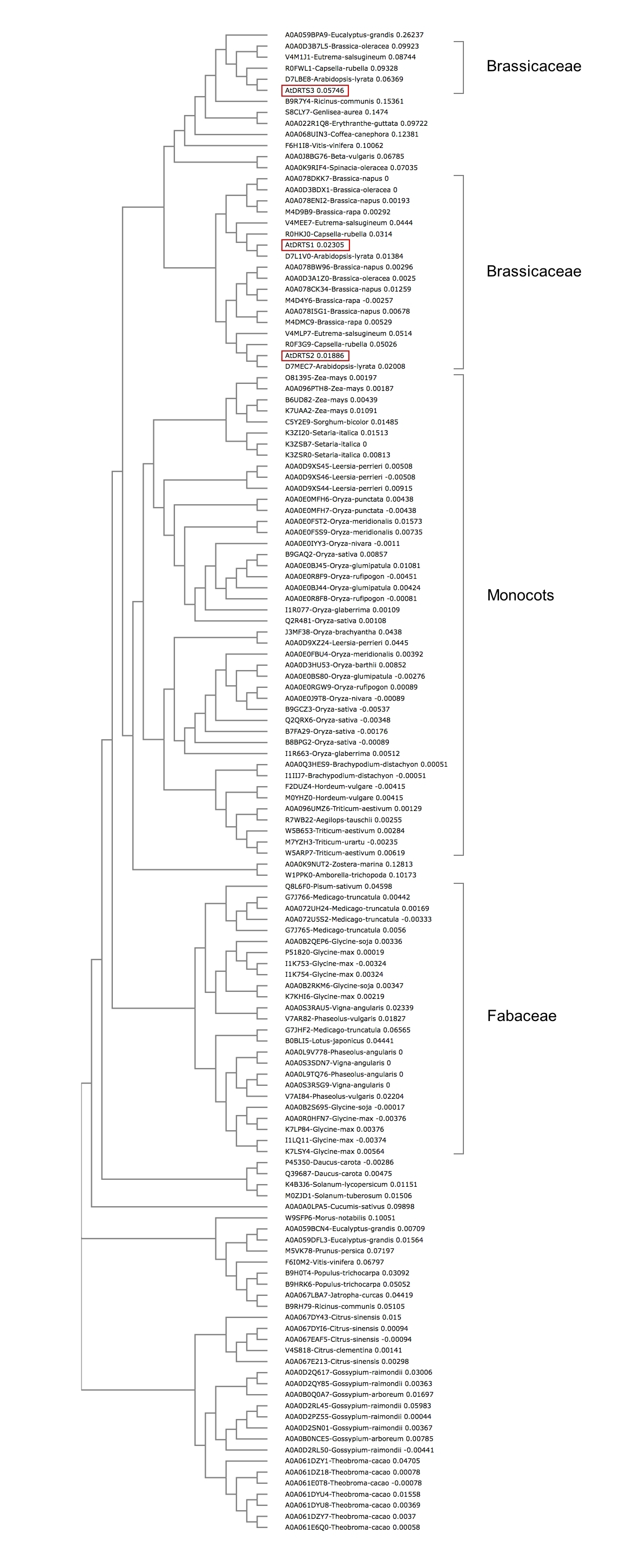

Supplement: S1 Fig — The Phylogenetic Tree was created aligning the aminoacid sequences with Clustal Omega (http://www.ebi.ac.uk/Tools/msa/clustalo/). The branches including monocots, Fabaceae and brassicaceae are pointed out. The AtDRTSs are indicated with red boxes. (TIF) [file pone.0179338.s001.tif]

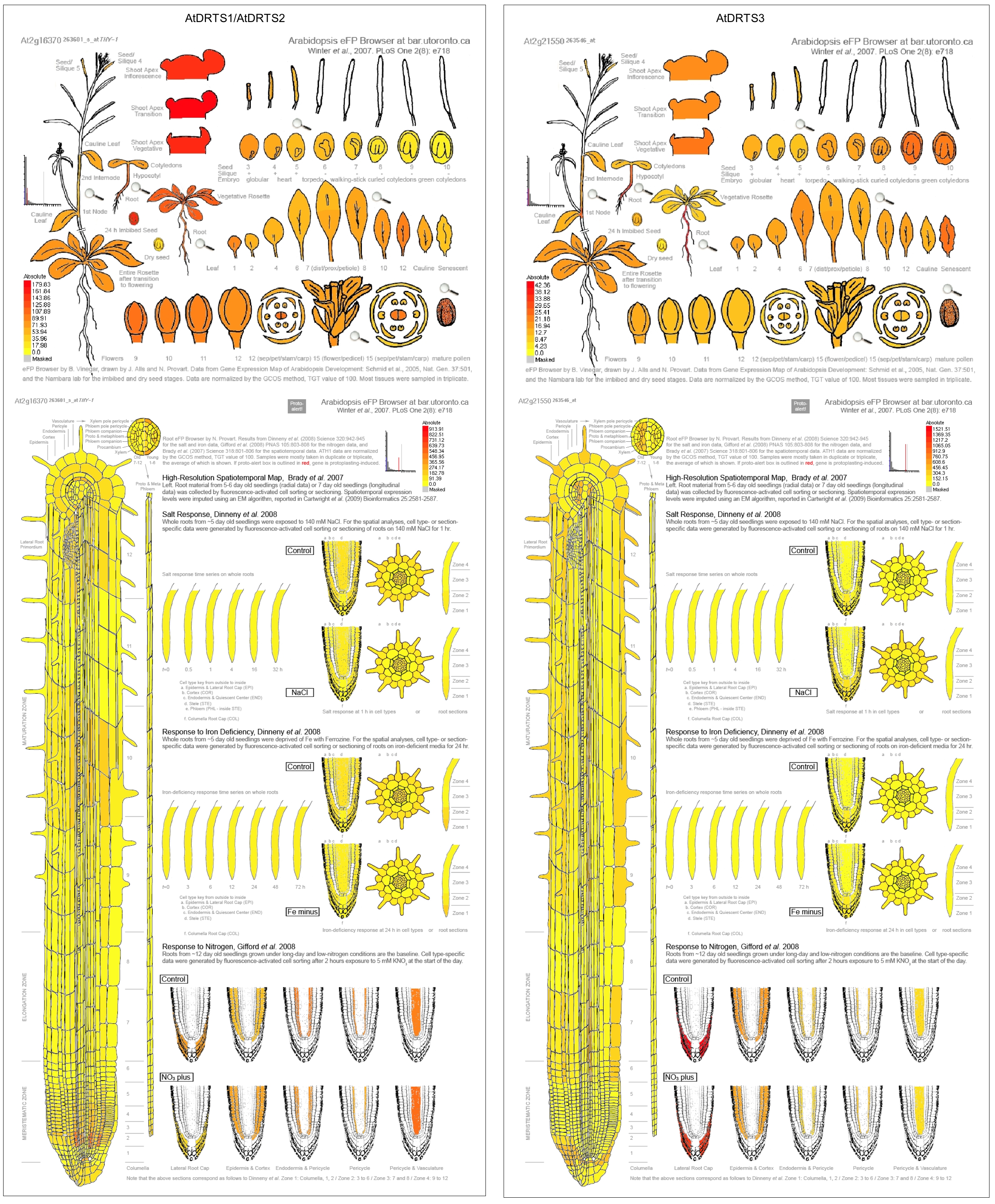

Supplement: S2 Fig — Data shown are reported at the Botany Array Resource (BAR) Browser (http://bar.utoronto.ca/). (TIF) [file pone.0179338.s002.tif]

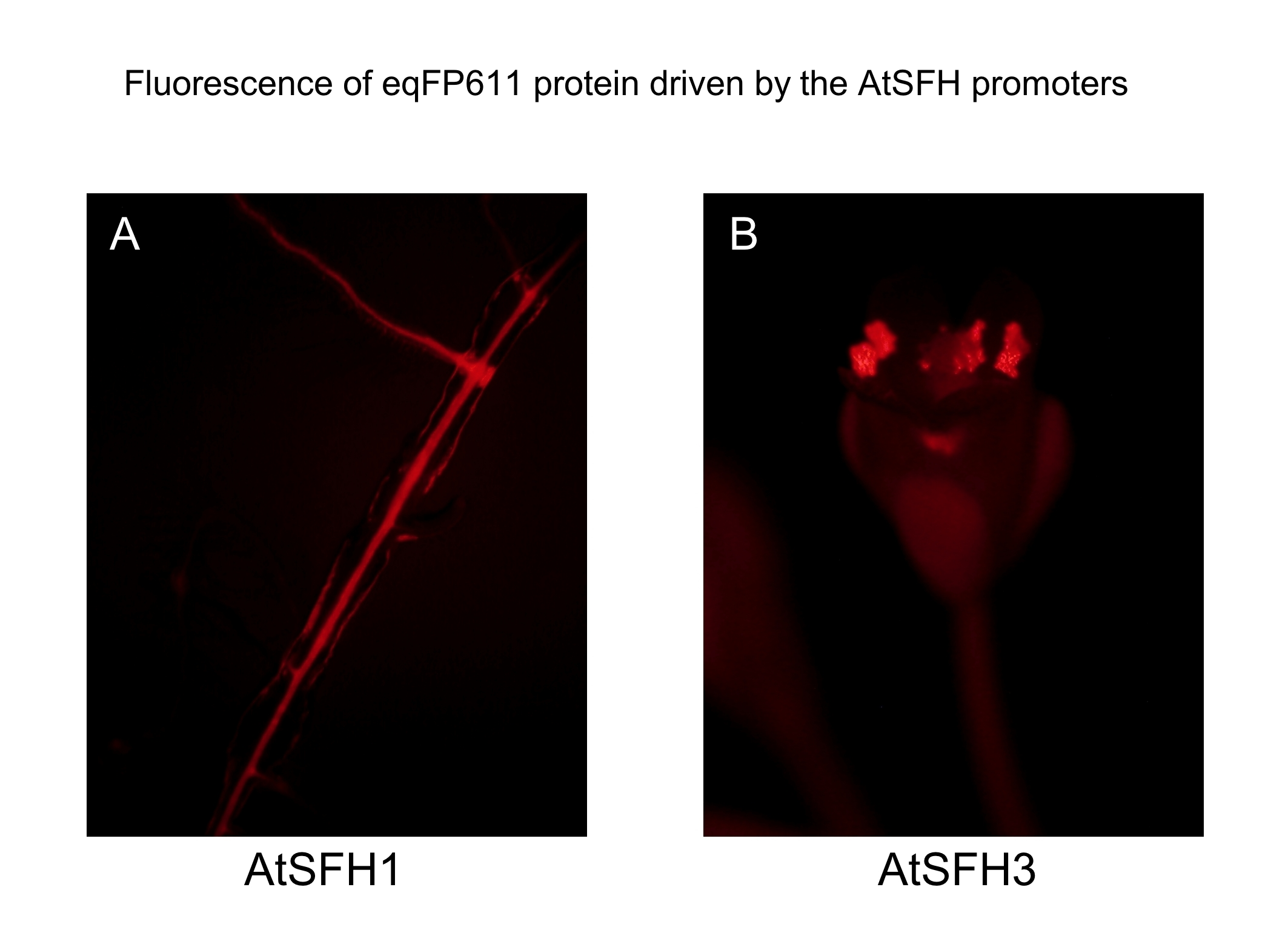

Supplement: S3 Fig — The fluorescence was visualized in vivo with a Wild M10 stereomicroscope equipped with a Leica fluorescence module using a standard TRITC filter set. (TIF) [file pone.0179338.s003.tif]
