# Supplementary material for: Distinctive features and differential regulation of the DRTS genes of Arabidopsis thaliana
Source: PLoS One. 2017 Jun 8;12(6):e0179338. doi: 10.1371/journal.pone.0179338 (PMC5464667; doi:10.1371/journal.pone.0179338)
Supplement: S1 Table — The restriction sites incorporated in the primers and used to clone the PCR fragments are underlined. (DOC) [file pone.0179338.s005.doc]

**S1 Table.** Primers used for PCR amplification and the assembling of the SFH/DRTS promoter constructs. The restriction sites are underlined.

| **Name** | **Sequence** |
| --- | --- |
| F16F2 | GTTGGATCCATTGTGGAAATCAAAACCTTG |
| F16F4 | GTCTCTAGAGGTTTAGACTTTTGATGAAAC |
| F16F5 | GGCGGATCCAATGCTTCCCTACACAAAT |
| T4L1 | TTTTCTAGATCTGGTTAGATGAGTTTTAAGCAC |
| T4L2 | TGTTTGCCATGGTTGAAATTGAAACCTTGA |
| T4L6 | TCTGAATTCGTTTATACCCTCTCCGAAGC |
| T4L7 | AACGAATTCAGACGACGGCGACTGAGTCA |
| F2G1 | ATCCTGCAGGGTTAAAGTCTGGATTTAAGATTT |
| F2G2 | CAGCCATGGCCATATTCTGAAACTTAA AAA TC |
| F2G3 | CGTGAATTCAACCCGTCCGTAAAAAACTAT |
| F2G4 | GTTGAATTCACGAGTTTGACAGGAAGTTAC |
| M13FW | ACGTTGTAAAACGACGGC |
| M13RV | GGAAACAGCTATGACCATG |
