# Supplementary material for: Distinctive features and differential regulation of the DRTS genes of Arabidopsis thaliana
Source: PLoS One. 2017 Jun 8;12(6):e0179338. doi: 10.1371/journal.pone.0179338 (PMC5464667; doi:10.1371/journal.pone.0179338)
Supplement: S2 Table — (DOC) [file pone.0179338.s006.doc]

**S2 Table.** Primers used for qRT-PCR analysis.

| **Name** | **Sequence** |
| --- | --- |
| RT-DRTS1-F | AAGTGTCGCCATTGAAATCC |
| RT-DRTS1-R | GCGAGTTTTCTGGAGAGGTG |
| RT-DRTS2-F | GAACAAGATCGCAGACGTGA |
| RT-DRTS2-R | ATGCCACATGTTTGCACAGT |
| RT-DRTS3-F | CACATGGCACGCTTATATCG |
| RT-DRTS3-F | TCTAGCTGCCACAACATTGC |
| RT-18S-F | CCTGCGGCTTAATTTGACTC |
| RT-18S-R | TTAGCAGGCTGAGGTCTCGT |
