# Supplementary material for: Distinctive features and differential regulation of the DRTS genes of Arabidopsis thaliana
Source: PLoS One. 2017 Jun 8;12(6):e0179338. doi: 10.1371/journal.pone.0179338 (PMC5464667; doi:10.1371/journal.pone.0179338)
Supplement: S3 Table — The analyses were performed searching against the PLACE (http://www.dna.affrc.go.jp/PLACE/), PlantPAN (http://plantpan2.itps.ncku.edu.tw/) and JASPAR (http://jaspar.genereg.net/) databases. The distance from the AtDRTSs ATG codon is reported. Sites that are closer to the AtDRTS coding region than to AtSFH are indicated in red. Cis elements related to expression in proliferating cells, endosperm expression and hormone response are highlighted in different colors. (DOC) [file pone.0179338.s007.doc]

**S3 table.** Presence and location of *cis* elements in the SFH/DRTS intergenic regions.

The analyses were performed searching against the PLACE (<http://www.dna.affrc.go.jp/PLACE/>), PlantPAN (<http://plantpan2.itps.ncku.edu.tw/>) and JASPAR (<http://jaspar.genereg.net/>) databases. The distance from the *AtDRTSs* ATG codon is reported. Sites that are closer to the *AtDRTS* coding region than to *AtSFH* are indicated in red. *Cis* elements related to expression in proliferating cells, endosperm expression and hormone response are highlighted in different colors.

| **CIS element** | **Sequence** | **AtDRTS1** | **AtDRTS2** | **AtDRTS3** |
| --- | --- | --- | --- | --- |
| **OSE1ROOTNODULE** | AAAGAT | -1607, -1497, -1046,  -959, -520, -216 | -1244, -1141 | -2395, -384, -89 |
| **MYBST1** | GGATA | -1061, -974, -718,  -465, -454, -122 | -543 | -2891, -912 |
| **REALPHALGLHCB21** | AACCAA | -1372, -925, -837,  -800, -730 | -633, -592 | -2985, -1022, -634,  -570, -20 |
| **PYRIMIDINEBOXOSRAMY1A** | CCTTTT | -903, -314, -163 | -313 | -3350, -2961, -2879,  -2819, -2801, -645 |
| **DPBFCOREDCDC3** | ACACNNG | -1554, -1342, -92 | -372 | -2709, -865, -666,  -270 |
| **MYBCORE** | CNGTTR | -810, -773 | -293 | -3442, -2562, -2358,  -2096, -1006, -121, -55 |
| **RAV1AAT** | CAACA | -559 | -1254, -784 | -3442, -2059, -1771,  -1556, -1002, -52 |
| **SURECOREATSULTR11** | GAGAC | -393, -75 | -204, -143 | -3287, -2010, -1232 |
| **CCA1ATLHCB1** | AAMAATCT | -1594, -631 | -1075, -979 | -1320, -1290, -512 |
| **SITEIIATCYTC** | TGGGCY | -1238 | -286 | -1116, -1054, -1043 |
| **CCA1-B** | AGATAYR | -1302 | -492 | -2392, -238 |
| **MYCATERD1** | CATGTG | -1343, -688 | -524, -372 | -667 |
| **-300CORE** | TGTAAAG | -128, -179 | -1246 | -386 |
| **PREATPRODH** | ACTCAT | -1524 | -1300, -1175 | -823 |
| **ACGTATERD1** | ACGT | -389 | -156 | -799, -213 |
| **SEF1MOTIF** | ATATTTAWW | -591 | -835, -625 | -1384, -1302 |
| **SEF3MOTIFGM** | AACCCA | -257 | -747 | -3204 |
| **LTRE1HVBLT49** | CCGAAA | -900, -713, -663 | -301 |  |
| **EECCRCAH1** | GANTTNC | -868, -582, -83,  -44, -4 | - | -3327, -2857, -2524,  -2500, -715, -452 |
| **NTBBF1ARROLB** | ACTTTA | -1230, -1181, -377 | - | -3463, -1856, -1035,  -790, -655, -244 |
| **SP8BFIBSP8BIB** | TACTATT | -625, -603 | - | -2566, -2452, -1242,  -840 |
| **GT1CORE** | GGTTAA | -802 | - | -3466, -3413, -1791,  -1720, -1134, -632 |
| **CIACADIANLELHC** | CAANNNNATC | -126 | - | -3223, -1408, -1025,  -396 |
| **PYRIMIDINEBOXHVEPB1** | TTTTTTCC | -1544 | - | -2803, -878, -831 |
| **AACACOREOSGLUB1** | AACAAAC | -1368 | - | -2336, -1865, -1268 |
| **LTRECOREATCOR15** | CCGAC | -430 | - | -2182, -1984, -347 |
| **ARFAT** | TGTCTC | -392 | - | -3286, -2010 |
| **GAREAT** | TAACAAR | -1428 | - | -3192, -1864 |
| **CATATGGMSAUR** | CATATG | -108 | - | -673, -446 |
| **LECPLEACS2** | TAAAATAT | -1162 | - | -2588, -1640 |
| **IBOX** | GATAAG | -1417, -332 | - | -1910 |
| **WBOXNTCHN48** | CTGACY | -189, -99 | - | -718 |
| **CANBNNAPA** | CNAACAC | -89 | - | -543 |
| **S1FBOXSORPS1L21** | ATGGTA | -1493 | - | -2622 |
| **SV40COREENHAN** | GTGGWWHG | -464 | - | -435 |
| **MYBPLANT** | MACCWAMC | -728 | - | -3110 |
| **CBFHV** | RYCGAC | - | -774, -268, --69 | -1984, -959, -891,  -347 |
| **CPBCSPOP** | TATTAG | - | -811, -388 | -3050, -2569, -2403 |
| **MYB2CONSENSUSAT** | YAACKG | - | -293 | -2562, -2096, -121,  -55 |
| **SORLIP2AT** | GGGCC | - | -310, -287 | -1116, -1055, -1044 |
| **NAPINMOTIFBN** | TACACAT | - | -672, -373 | -665 |
| **RBCSCONSENSUS** | AATCCAA | - | -915 | -3385, -2854, -805,  -27 |
| **MYBPZM** | CCWACC | - | -1263 | -3152, -3112, -1590 |
| **TBOXATGAPB** | ACTTTG | - | -330 | -2212, -1737, -1684 |
| **BOXLCOREDCPAL** | ACCWWCC | - | -1263 | -3111, -333 |
| **MYBCOREATCYCB1** | AACGG | - | -196 | -2700, -2165 |
| **-10PEHVPSBD** | TATTCT | - | -345 | -1873 |
| **UP1ATMSD** | GGCCCAWWW | - | -283 | -1040 |
| **E2F-LIKE** | NNTSSCGSS | - | -199 | -1591 |
| **TATCCAOSAMY** | TATCCA | -1061, -974, -465, -122 | - | - |
| **ASF1MOTIFCAMV** | TGACG | -1262, -1108 | - | - |
| **EMHVCHORD** | TGTAAAGT | -1228, -1179 | - | - |
| **BOXIINTPATPB** | ATAGAA | -1274, -410 | - | - |
| **SREATMSD** | TTATCC | -1060, -973 | - | - |
| **AMMORESIIUDCRNIA1** | GGWAGGGT | -1504 | - | - |
| **SURE1STPAT21** | AATAGAAAA | -1272 | - | - |
| **MYC2 ELEMENT** | TCACATG | -688 | - | - |
| **TATCCACHVAL21** | TATCCAC | -465 | - | - |
| **REBETALGLHCB21** | CGGATA | -454 | - | - |
| **CAREOSREP1** | CAACTC | -308 | - | - |
| **ABRE-LIKE** | BACGTGKM | -388 | - | - |
| **GADOWNAT** | ACGTGTC | -389 | - | - |
| **BS1EGCCR** | AGCGGG | -336 | - | - |
| **NRRBNEXTA** | TAGTGGAT | -466 | - | - |
| **2SSEEDPROTBANAPA** | CAAACAC | -89 | - | - |
| **BOXIINTPATPB** | ATAGAA | - | -937 | - |
| **MYB1LEPR** | GTTAGTT | - | -852 | - |
| **GCN4OSGLUB1** | TGAGTCA | - | -218 | - |
| **HEXAMERATH4** | CCGTCG | ~~-~~ | -208 | ~~-~~ |
| **UP2ATMSD TELOBOXATEEF1AA1** | AAACCCTA | - | -130 | - |
| **CCA1-A** | AATATCY | - | - | -2889, -2684, -1478 |
| **SEBFCONSSTPR10A** | YTGTCWC | - | - | -3438, -2010 |
| **AMYBOX1** | TAACARA | - | - | -3192, -1864 |
| **CTRMCAMV35S** | TCTCTCTCT | - | - | -2906, -2845 |
| **ELRECOREPCRP1** | TTGACC | - | - | -2794, -1773 |
| **ERELEE4** | AWTTCAAA | - | - | -2208, -1890 |
| **MARABOX1** | AATAAAYAAA | - | - | -3353, -2128 |
| **MYBGAHV** | TAACAAA | - | - | -3192, -1864 |
| **P1BS** | GNATATNC | - | - | -3135, -2997 |
| **QELEMENTZMZM13** | AGGTCA | - | - | -2973, -1774 |
| **PALBOXAPC** | CCGTCC | - | - | -2957, -1585 |
| **CGCGBOXAT** | VCGCGB | - | - | -2711, -1715 |
| **HDZIP2ATATHB2** | TAATMATTA | - | - | -2238, -845 |
| **ANAERO2CONSENSUS** | AGCAGC | - | - | -943, -760 |
| **WUSATAg** | TTAATGG | - | - | -2823 |
| **WBBOXPCWRKY1** | TTTGACY | - | - | -2794 |
| **ABRERATCAL** | MACGYGB | - | - | -2710 |
| **D1GMAUX28** | ACAGTTACTA | - | - | -2561 |
| **L1BOXATPDF1** | TAAATGYA | - | - | -2364 |
| **SORLIP1AT** | GCCAC | - | - | -1252 |
| **SORLIP5AT** | GAGTGAG | - | - | -825 |
| **RHERPATEXPA7** | KCACGW | - | - | -797 |
| **RYREPEATLEGUMINBOX** | CATGCAY | - | - | -669 |

Legend:

**CELL PROLIFERATION**

**AUXIN**

**CYTOCHININ**

**GIBBERELLIN**

**ABA**

**ETHYLENE**

**JASMONATE**

**ENDOSPERM**
