# Supplementary material for: Distinctive features and differential regulation of the DRTS genes of Arabidopsis thaliana
Source: PLoS One. 2017 Jun 8;12(6):e0179338. doi: 10.1371/journal.pone.0179338 (PMC5464667; doi:10.1371/journal.pone.0179338)
Supplement: S4 Table — The analyses were performed searching against the PLACE (http://www.dna.affrc.go.jp/PLACE/), PlantPAN (http://plantpan2.itps.ncku.edu.tw/) and JASPAR (http://jaspar.genereg.net/) databases. Position from ATG corresponds to the bp distance downstream from the ATG codon. Sites located within the second intron of the AtDRTS1 gene are shown in red. (DOC) [file pone.0179338.s008.doc]

**S4 table.** Presence and location of *cis* elements in the intragenic 5’ region of *AtDRTS1* that includes the second intron of the gene.

The analyses were performed searching against the PLACE (<http://www.dna.affrc.go.jp/PLACE/>), PlantPAN (<http://plantpan2.itps.ncku.edu.tw/>) and JASPAR (<http://jaspar.genereg.net/>) databases.

Position from ATG corresponds to the bp distance downstream from the ATG codon.

Sites located within the second intron of the *AtDRTS1* gene are shown in red.

| **CIS element** | **Sequence** | **Position from ATG** |
| --- | --- | --- |
| **DPBFCOREDCDC3** | ACACNNG | 34 |
| **TBOXATGAPB** | ACTTTG | 160 |
| **ELRECOREPCRP1** | TTGACC | 163 |
| **GT1GMSCAM4** | GAAAAA | 186 |
| **LTRE1HVBLT49** | CCGAAA | 248 |
| **LTRECOREATCOR15** | CCGAC | 254 |
| **ACGTATERD1** | ACGT | 263 |
| **SEF3MOTIFGM** | AACCCA | 285 |
| **GAREAT** | TAACAAR | 348, 391 |
| **SREATMSD** | TTATCC | 444 |
| **MYBST1** | GGATA | 445 |
| **REALPHALGLHCB21** | AACCAA | 475, 492, 496 |
| **MYBPLANT** | MACCWAMC | 494 |
| **BOXLCOREDCPAL** | ACCWWCC | 494 |
