# Supplementary material for: Distinctive features and differential regulation of the DRTS genes of Arabidopsis thaliana
Source: PLoS One. 2017 Jun 8;12(6):e0179338. doi: 10.1371/journal.pone.0179338 (PMC5464667; doi:10.1371/journal.pone.0179338)
Supplement: S5 Table — The accumulation of the AtE2Fa mRNA in one-week-old seedlings was quantified by qPCR following the ΔΔCt method using the18S RNA as a reference for normalization. The qPCR analysis was carried out using three biological replicates. The mean level of expression with the SE is reported. The phenotypic analysis of the cotyledons was carried out on 12-day-old plants using 8 to 12 samples. The size of the adaxial epidermal cells was calculated counting the number of cells contained in an area of 100,000 μm2. The total epidermal cell number was estimated dividing the cotyledon size by the cell size. The mean values with the SE are reported. (DOC) [file pone.0179338.s009.doc]

S5 Table. Features of the two Arabidopsis lines overexpressing the AtE2Fa factor.

|  | AtE2Fa^OE^ #1 | AtE2Fa^OE^ #5 | Wild Type |
| --- | --- | --- | --- |
| Fold overexpression | 113.7 ± 11.8 | 158.4 ± 12.7 | - |
| Cotyledon size (mm^2^) | 4.4 ± 0.5 | 5.4 ± 0.3 | 3.1 ± 0.2 |
| Epidermal cell size (μm^2^) | 2976.2 ± 106.3 | 3215.4 ± 175.8 | 5494.5 ± 211.3 |
| Epidermal cell number | 1478.4 ± 168.0 | 1679.4 ± 93.3 | 564.2 ± 36.4 |

The accumulation of the *AtE2Fa* mRNA in one-week-old seedlings was quantified by qPCR following the ΔΔ^Ct^ method using the18S RNA as a reference for normalization. The qPCR analysis was carried out using three biological replicates. The mean level of expression with the SE is reported. The phenotypic analysis of the cotyledons was carried out on 12-day-old plants using 8 to 12 samples. The size of the adaxial epidermal cells was calculated counting the number of cells contained in an area of 100,000 μm^2^ . The total epidermal cell number was estimated dividing the cotyledon size by the cell size. The mean values with the SE are reported.
